# Catalytic Transformation of Ginsenoside Re over Mesoporous Silica-Supported Heteropoly Acids: Generation of Diverse Rare Ginsenosides in Aqueous Ethanol Revealed by HPLC-HRMS^n^

**DOI:** 10.3390/molecules30244753

**Published:** 2025-12-12

**Authors:** Qi Wang, Yanyan Chang, Bing Li, Zhenxuan Zhang, Mengya Zhao, Huanxi Zhao, Yang Xiu

**Affiliations:** Jilin Ginseng Academy, Changchun University of Chinese Medicine, Changchun 130117, China; 13278542346@163.com (Q.W.);

**Keywords:** rare ginsenosides, heterogeneous transformation, heteropolyacid, mesoporous silica, mass spectrometry

## Abstract

The efficient generation of structurally diverse rare ginsenosides from abundant precursors remains a significant challenge. In this study, a heterogeneous catalyst, 12-tungstosilicic acid supported on mesoporous silica (HSiW@mSiO_2_), was developed for the transformation of ginsenoside Re in aqueous ethanol solution. The reaction was conducted under mild conditions, and the products were systematically analyzed using high-performance liquid chromatography coupled with multistage tandem mass spectrometry and high-resolution mass spectrometry. A total of 24 transformation products were identified, arising from deglycosylation, epimerization, dehydration, cyclization, and nucleophilic addition reactions. Structural elucidation revealed the formation of deglycosylated, hydrated and dehydrated derivatives, C-20 epimers, and novel ethoxylated protopanaxatriol-type ginsenosides resulting from solvent incorporation at the C-24(25) or C-20 position. Product distribution varied with reaction parameters, including solvent composition, reaction time, temperature, and catalyst dosage. The synthesized HSiW@mSiO_2_ catalyst could be readily recovered by centrifugation and reused for five consecutive cycles, with complete conversion of ginsenoside Re maintained in the first two runs and a gradual decline in conversion to approximately 50% by the fifth cycle. This work demonstrates the efficacy of solid acid catalysts in enabling the structural diversification of ginsenosides through solvent-involved pathways.

## 1. Introduction

**Figure 1:**
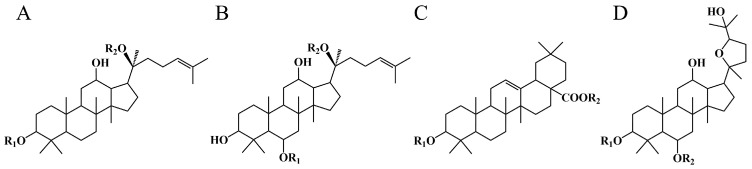
Chemical structures of PPD-type (**A**), PPT-type (**B**), ocotillol-type (**C**), and oleanolic acid-type (**D**) ginsenosides.

Ginseng (*Panax ginseng* C. A. Meyer), with its prolonged growth cycle and remarkable medicinal properties, is regarded as a unique and highly valued herb in traditional Chinese medicine [1,2]. The chemical constituents of ginseng comprise ginsenosides, volatile components, polysaccharides, amino acids and peptides, trace elements, and other minor constituents [3]. Among these, ginsenosides have been identified as the primary bioactive components mediating its pharmacological effects [4]. As shown in Figure 1, ginsenosides are categorized into four structural types based on their aglycone skeletons: protopanaxadiol (PPD)-type, protopanaxatriol (PPT)-type, ocotillol-type, and oleanolic acid-type [5]. Additionally, they are classified into major ginsenosides and rare ginsenosides according to their natural abundance. Rare ginsenosides typically exhibit superior bioavailability and more targeted bioactivities compared to their major counterparts. For instance, the anticancer efficacy of ginsenoside Rg3 and the anti-inflammatory activity of ginsenoside Rh2 have been extensively validated through preclinical and clinical investigations [6,7,8]. However, the naturally low abundance of rare ginsenosides, coupled with the prohibitively high costs of their extraction and purification processes, severely limits their large-scale application. Structural characterization of rare ginsenosides has revealed that their main structural differences from major ginsenosides involve a reduced number of glycosyl substituents and modified skeletons. Consequently, the hydrolysis and modification of major ginsenosides, which reduce glycosylation and alter skeletal structures, represent a viable strategy for the production of diverse rare ginsenosides.

The C-24(25) double bond in PPT and PPD-type ginsenosides serves as a key site for structural diversification. This alkene chain can undergo acid-catalyzed electrophilic addition, forming a C-25 carbocation intermediate that is susceptible to nucleophilic attack [9]. In aqueous alcoholic systems, both water and the alcohol (e.g., methanol, ethanol) can act as nucleophiles, leading to competitive hydration or alkoxy addition at C-25, thereby generating 25-hydroxylated and 25-alkoxyl derivatives, respectively. These 25-functionalized rare ginsenosides are scarcely found in nature and represent valuable structural analogs [10,11,12]. Notably, biotransformation studies demonstrate that microorganisms can similarly target this site via enzymatic hydroxylation, producing novel rare ginsenosides from Rb1 [13]. This convergence of chemical and biological pathways underscores the C-24(25) double bond as a critical “hotspot” for generating structural diversity. The resulting hydroxylation and alkoxylation at C-25 is associated with enhanced pharmacological activities in these derivatives compared to their parent compounds. Therefore, an ideal catalytic system should enable not only glycosidic bond cleavage but also versatile functionalization of the aglycone skeleton.

The preparation of rare ginsenosides currently relies on either biotransformation or chemical transformation of major ginsenosides [9,14]. Chemical transformation, which typically requires strong acids and heavy metal catalysts, poses environmental risks and complicates subsequent product purification. In contrast, enzymatic catalysis, while benefiting from mild reaction conditions, also faces challenges such as high production costs and insufficient catalyst stability. In response to these issues, heteropoly acids (HPAs) offer a promising approach for the efficient transformation of ginsenosides owing to their unique acidic and redox properties [15]. HPAs represent a class of nanoscale cluster compounds comprising transition metals (e.g., tungsten and molybdenum) and oxygen atoms. These compounds feature strong Brønsted and Lewis acid sites that enable precise catalysis of glycosidic bond hydrolysis reactions. Furthermore, their stable cage structures (e.g., Keggin-type and Dawson-type) confer excellent reusability [16].

However, the primary limitations of HPAs in catalytic applications include their small specific surface area and poor solubility in polar solvents, factors that not only limit catalytic activity but also complicate separation from the reaction mixture. Mesoporous silica (mSiO_2_) is an excellent support for HPAs due to its highly ordered pore structure with a tunable pore size ranging from 2 nm to 50 nm, high specific surface area, and abundant surface silanol groups [17]. The large specific surface area and uniform pore size distribution of mSiO_2_ facilitate the highly dispersed immobilization and spatial confinement of HPA nanoclusters, thereby significantly increasing the density of catalytic active sites. Furthermore, the surface silanol groups can anchor HPAs through hydrogen bonding or covalent linkages, thereby effectively mitigating the leaching of active species. Recent studies have demonstrated distinct strategies to immobilize Keggin-type HPAs on silica supports. Falcão et al. used an in situ sol–gel co-condensation approach to embed tungstophosphoric acid directly into a silica matrix, preserving the Keggin structure and enhancing acidity for dehydration reactions [18]. In contrast, Zhang et al. immobilized tungstophosphoric acid onto a pre-formed Fe_3_O_4_@SiO_2_@TiO_2_ support via post-synthetic impregnation, yielding a magnetically recoverable catalyst that exhibited exceptional performance in the synthesis of 5-hydroxymethylfurfural from fructose and inulin, along with excellent recyclability over at least five consecutive cycles [19]. These examples demonstrate that effective immobilization on mSiO_2_ can improve the catalyst stability and reusability of HPAs. Therefore, mSiO_2_-supported HPA catalysts are expected to serve as an efficient platform for the comprehensive transformation of major ginsenosides, enabling not only deglycosylation but also diverse functionalization.

High-performance liquid chromatography–mass spectrometry (HPLC-MS) offers significant advantages in the analysis of ginsenosides [20,21,22]. This technique facilitates the efficient separation and highly specific identification of complex transformation products by combining high-resolution mass spectrometry (HRMS) and multistage tandem mass spectrometry (MS^n^). HRMS enables precise compound identification by providing high-accuracy mass measurement [23]. MS^n^ enables the accurate elucidation of molecular structures and the differentiation of isomers via the sequential selection and fragmentation of precursor ions, thereby generating comprehensive multistage fragmentation data [24].

In the present study, 12-tungstosilicic acid (H_4_SiW_12_O_40_, HSiW) was immobilized on a mSiO_2_ framework to synthesize the heterogeneous catalyst HSiW@mSiO_2_, which was subsequently used for the catalytic transformation of ginsenoside Re in an aqueous ethanol solution. The transformation products were effectively separated and structurally characterized using HPLC-MS^n^ and HRMS analyses. A total of 24 rare ginsenosides were successfully obtained from ginsenoside Re through a series of reactions, including deglycosylation, addition, elimination, cyclization, and epimerization. In addition, the reusability of the heterogeneous catalyst HSiW@mSiO_2_ was evaluated over multiple catalytic cycles in ginsenoside transformation reactions.

## 2. Results and Discussion

### 2.1. Characterization of HSiW@mSiO_2_

**Figure 2:**
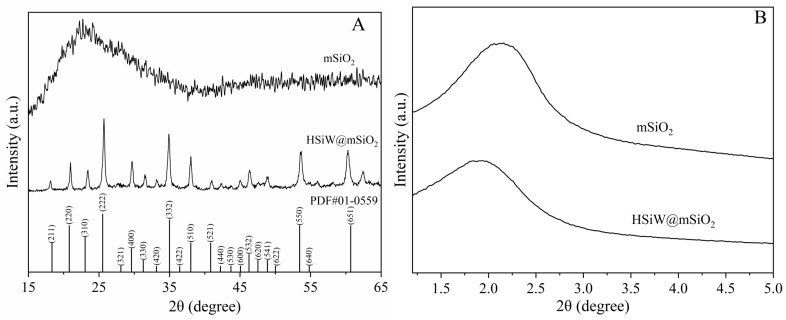
XRD patterns of mSiO_2_ and HSiW@mSiO_2_ over the 2θ range of 15–65° (**A**) and 1.2–5° (**B**).

The immobilization of HSiW on the mSiO_2_ framework was first confirmed through combined wide-angle and small-angle powder X-ray diffraction (XRD). In the wide-angle XRD pattern (Figure 2A), the as-prepared mSiO_2_ framework exhibits a broad peak centered at 23.8°, indicating its amorphous structure. After HSiW immobilization, characteristic diffraction peaks at 25.2°, 34.7°, 53.2°, and 61.4° appear in the HSiW@mSiO_2_ composite, corresponding to the (222), (332), (550), and (651) crystallographic planes of the Keggin-type HSiW (PDF#01-0559). The appearance of characteristic diffraction peaks of HSiW in the XRD pattern of HSiW@mSiO_2_ suggests that the Keggin-type structure of HSiW is retained after immobilization. The presence of sharp reflections further indicates that HSiW is present as nanocrystalline domains rather than being molecularly dispersed within the mSiO_2_ matrix [25]. As shown in Figure 2B, the pristine mSiO_2_ exhibits a distinct low-angle diffraction peak at 2.1°, corresponding to the (100) reflection of a hexagonally ordered mesoporous structure, indicating the presence of uniform and periodic pore channels. After HSiW loading, the peak shifts slightly to 1.9°, indicating a minor increase in the interplanar spacing of the framework [26]. This suggests that the long-range order is maintained, while HSiW incorporation induces a subtle lattice expansion, likely due to interactions between the heteropoly acid and the pore walls. Therefore, XRD analysis confirms the successful preparation of HSiW@mSiO_2_, with retention of both the Keggin structure of HSiW and the ordered mesoporous framework of mSiO_2_.

As shown in Figure S1, the Fourier-transform infrared (FTIR) spectrum of the HSiW@mSiO_2_ composite clearly displays the characteristic vibrational modes associated with both the mSiO_2_ framework and the immobilized HSiW, indicating that neither component undergoes structural alteration during immobilization. In the composite, the vibrational characteristics of Keggin-type HSiW are distinctly observed: the band at 980 cm^−1^ is assigned to the terminal W=O stretching vibration, and the peak at 930 cm^−1^ to the central Si-O stretching vibration. Additionally, the adsorption bands at 883 cm^−1^ and 800 cm^−1^ arise from W-O-W bridges formed by corner- and edge-sharing oxygen atoms between W_3_O_13_ units, respectively [27,28,29], confirming the integrity of the Keggin structure. The broad band between 1300 cm^−1^ and 1000 cm^−1^ is attributed to the asymmetric stretching vibration of Si-O-Si linkages, while the bands at 803 cm^−1^ and 465 cm^−1^ correspond to the symmetric stretching and bending vibrations of the same bonds, respectively [30,31,32]. These features confirm the retention of the silica-based mesoporous framework. Notably, no significant peak broadening, shifting, or disappearance is observed, suggesting that the chemical interactions are extremely weak, insufficient to distort the HSiW structure, while still enabling stable immobilization on the mSiO_2_ framework.

**Figure 3:**
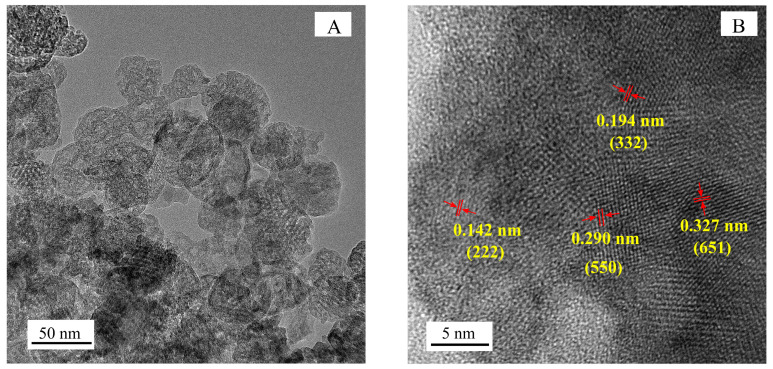
TEM (**A**) and HRTEM (**B**) images of HSiW@mSiO_2_.

Transmission electron microscopy (TEM) images further provide direct evidence of HSiW incorporation on the mSiO_2_ framework. Figure 3A reveals that the spherical mSiO_2_ particles, with an average diameter of approximately 50 nm, retain their ordered mesoporous structure following HSiW immobilization. As shown in Figure 3B, well-defined lattice fringes are observed from the HSiW@mSiO_2_ composite, with measured interplanar spacings of 0.142 nm, 0.194 nm, 0.290 nm, and 0.327 nm, corresponding to the (222), (332), (550), and (651) crystallographic planes of the Keggin-type HSiW, respectively. This confirms the preservation of its crystalline structure upon immobilization. The coexistence of distinct lattice fringes and intact spherical morphology indicates that HSiW is structurally integrated with the mSiO_2_ framework, forming a well-structured hybrid composite. Furthermore, the synthesized HSiW@mSiO_2_ composite exhibits excellent insolubility in aqueous ethanol solution, a crucial requirement for heterogeneous catalysis. The combination of structural integrity and phase stability reveals the potential of HSiW@mSiO_2_ as a catalyst for the heterogeneous transformation of ginsenosides [33,34]. Notably, although silica-supported HSiW catalysts prepared by impregnation or hybrid sol–gel strategies have been used in acid-catalyzed reactions such as alcohol dehydration and alkylation [18,19], their use in the transformation of PPT-type ginsenosides in an aqueous ethanol environment has not been investigated.

### 2.2. Structural Characterization and Identification of Ginsenoside Re Transformation Products in Aqueous Ethanol by HPLC-MS

**Figure 4:**
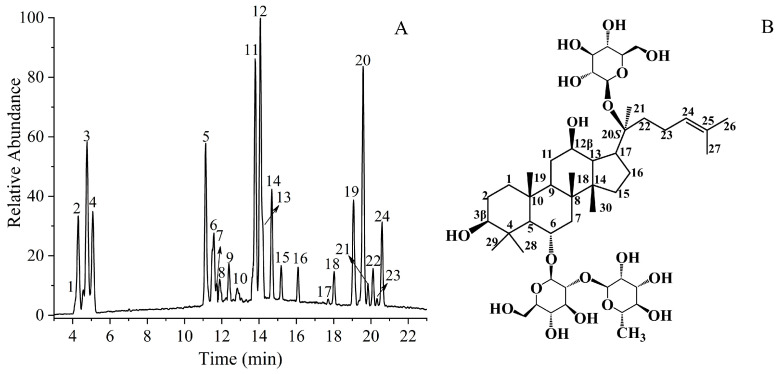
TIC of the transformation products of ginsenoside Re with ethanol (**A**) and the structural formula of ginsenoside Re (**B**).

The chemical transformation of ginsenoside Re was carried out using synthesized HSiW@mSiO_2_ as a catalyst in 70% aqueous ethanol at 80 °C for 4 h, and the resulting products were analyzed by HPLC-MS. The total ion chromatogram (Figure 4A) revealed 24 distinct compounds, designated as 1 to 24, all of which were subjected to comprehensive structural elucidation using MS^n^ and HRMS. For comparison, the TIC of untreated ginsenoside Re under identical HPLC-MS conditions is provided in Figure S2. The structure of the reactant ginsenoside Re is shown in Figure 4B, illustrating its glycosylation pattern: a disaccharide Glc(2-1)-Rha at C-6 and a glucosyl substituent at C-20, which serves as the reference framework for structural derivation of the transformation products. The structural identification for all 24 compounds was primarily based on accurate molecular masses from HRMS, characteristic fragmentation patterns in MS^n^ spectra, and retention time matching with authentic ginsenoside standards, collectively ensuring the reliability and consistency of each identification.

#### 2.2.1. Identification of Ethanol Adducts

**Figure 5:**
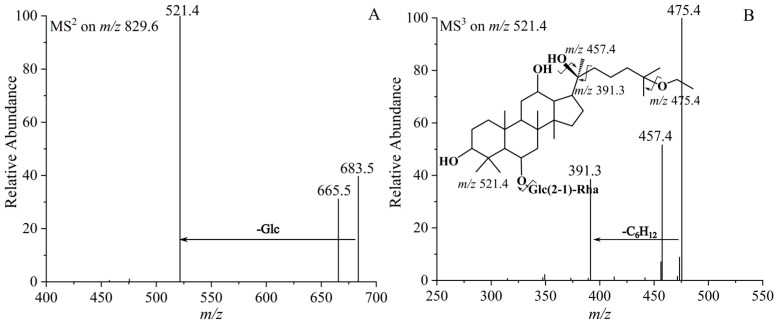
MS^2^ spectrum of the [M−H]^−^ ion at *m*/*z* 829.6 (**A**), fragmentation pathways, and MS^3^ spectrum of the product ion at *m*/*z* 521.4 (**B**) from ginsenoside (20*S*)-25-OCH_2_CH_3_-Rg2.

Compounds **8** and **9** were identified as a pair of isomers with the identical molecular formula of C_44_H_78_O_14_ and a relative molecular mass of 830.6. The MS^2^ spectrum of compound **8** on its [M−H]^−^ ion at *m*/*z* 829.6 (Figure 5A) exhibited product ions at *m*/*z* 683.5 and *m*/*z* 521.4, corresponding to sequential losses of rhamnosyl (146.1 Da) and glucosyl (162.1 Da) substituents. These fragments indicate that compound **8** retained the Glc(2-1)-Rha disaccharide substituent at the C-6 position, but lost the glucosyl substituent at the C-20 position.

Further MS^3^ analysis was performed on the product ion at *m*/*z* 521.4 (Figure 5B), which revealed a neutral loss of 46.0 Da, corresponding to the elimination of an ethanol molecule, yielding the ion at *m*/*z* 475.4. This suggests the presence of an ethoxyl group in the aglycone structure. The ion at *m*/*z* 475.4 is assigned to the characteristic deprotonated aglycone of PPT-type ginsenosides. Additionally, the presence of the product ion at *m*/*z* 391.3, along with an 84.1 Da neutral loss corresponding to the C_6_H_12_ molecule from the ion at *m*/*z* 475.4, indicates cleavage of the C-20(22) single bond in the olefin chain and preservation of the tetracyclic skeleton. This rules out the possibility of ethanol addition to the double bond formed at C-20 upon dehydration, as such dehydration would disrupt the olefin chain and prevent the observed fragmentation. Instead, it confirms that the ethoxyl group was preferentially attached to the C-24(25) double bond [11]. Therefore, compound 8 is derived from hydrolytic deglycosylation at the C-20 position, accompanied by ethanol addition to the C-24(25) double bond. Based on the chromatographic behavior of ginsenoside epimers on a C18 column, where (20*S*)-epimers exhibit higher polarity and elute earlier than their (20*R*)-counterparts [9,10,11,12,13,35,36], compounds **8** and **9** are assigned as (20*S*)-25-OCH_2_CH_3_-Rg2 and (20*R*)-25-OCH_2_CH_3_-Rg2, respectively. Their structures are illustrated in Figure 5B and Figure S3.

**Figure 6:**
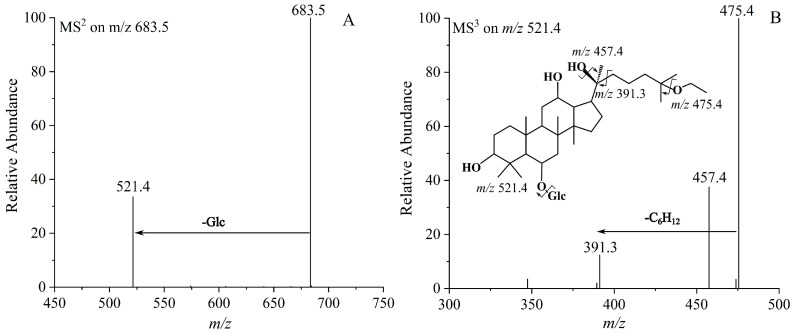
MS^2^ spectrum of the [M−H]^−^ ion at *m*/*z* 683.5 (**A**), fragmentation pathways, and MS^3^ spectrum of the product ion at *m*/*z* 521.4 (**B**) from ginsenoside (20*S*)-25-OCH_2_CH_3_-Rh1.

Compounds **7** and **10** were similarly identified as a pair of epimers, sharing an [M−H]^−^ ion at *m*/*z* 683.5, corresponding to a molecular formula of C_38_H_68_O_10_ and a relative molecular mass of 684.5. The MS^2^ spectra of their [M−H]^−^ ions in Figure 6A and Figure S4A exhibited a single neutral loss of 162.1 Da, derived from glucosyl elimination and generating the main product ion at *m*/*z* 521.4. This indicates that compounds **7** and **10** retained only a glucosyl substituent at the C-6 position. As shown in Figure 6B and Figure S4B, MS^3^ analysis of the ions at *m*/*z* 521.4 revealed product ions at *m*/*z* 475.4, *m*/*z* 457.4, and *m*/*z* 391.3, which are consistent with those of compounds **8** and **9**, suggesting the presence of the same ethoxylated PPT-type aglycone. Therefore, compounds **7** and **10** are derived from the further loss of the rhamnosyl substituent from compounds **8** and **9**. They are identified as (20*S*)-25-OCH_2_CH_3_-Rh1 and (20*R*)-25-OCH_2_CH_3_-Rh1, respectively.

**Figure 7:**
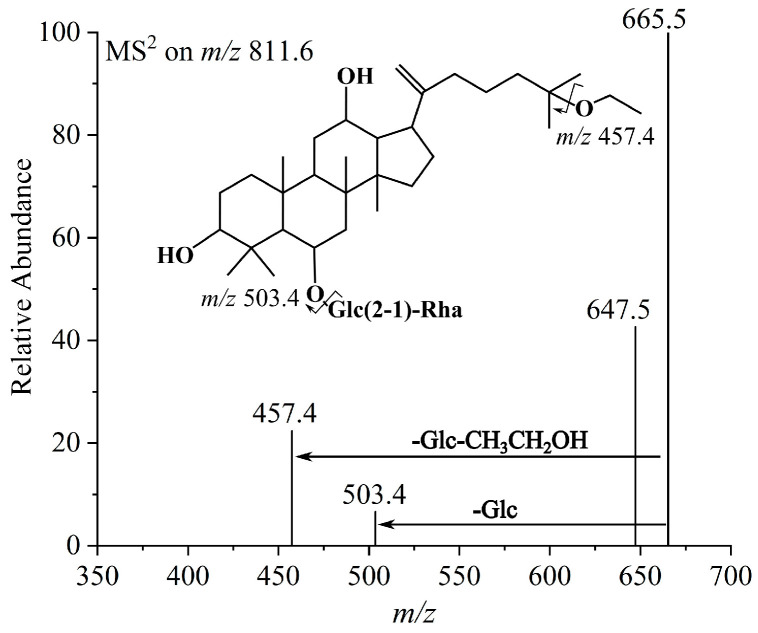
MS^2^ spectrum of the [M−H]^−^ ion at *m*/*z* 811.6 from ginsenoside 25-OCH_2_CH_3_-Rg6.

Compounds **17**, **18**, **21**, and **23** constitute a set of four isomeric ginsenosides with an identical molecular formula of C_44_H_76_O_13_ and a relative molecular mass of 812.6. They exhibit nearly identical MS^2^ spectra of their [M−H]^−^ ion at *m*/*z* 811.6, as shown in Figure 7, Figures S5 and S6. The product ions at *m*/*z* 665.5 and *m*/*z* 503.4, corresponding to sequential neutral loss of 146.1 Da and 162.1 Da, suggest the retention of the rutinose disaccharide substituent at the C-6 position. Given their relatively close retention times of compounds **17** and **18**, and compounds **21** and **23**, they are inferred to represent two pairs of structurally related isomers. Furthermore, the absence of the characteristic fragment at *m*/*z* 391.3 in all four compounds suggests a structural modification at the C-20 position.

A neutral loss of 46.0 Da between the aglycone ion at *m*/*z* 503.4 and the ion at *m*/*z* 457.4 indicates the presence of an ethoxyl group in the aglycone. The mass difference of 18.0 Da between the de-ethoxylated ion at *m*/*z* 457.4 and the absent characteristic aglycone ion of PPT-type ginsenosides at *m*/*z* 475.4 implies the formation of an additional double bond beyond the C-24(25) position. This extra unsaturation can only arise from dehydration of the hydroxyl group at C-20 following deglycosylation at this site. Subsequent addition of ethanol to these two double bonds at C-24(25) and C-20(21/22) would generate two pairs of ethoxylated isomers, consistent with the chromatographic pairing observed. According to Markovnikov’s rule under acidic conditions, when ethanol adds to the C-24(25) double bond, the ethoxyl group attaches to C-25, while the other double bond remains at C-20(21/22), forming Δ20(21) and Δ20(22) isomeric products. In contrast, when the addition occurs to the C-20(21/22) double bond, the ethoxyl group attaches to C-20, resulting in a pair of C-20 epimers and leaving the other double bond at C-24(25). Based on previous reports that 25-alkoxyl ginsenosides elute earlier than their 20-alkoxyl counterparts on C18 columns [12], compounds **17**, **18**, **21**, and **23** are therefore assigned as 25-OCH_2_CH_3_-Rg6, 25-OCH_2_CH_3_-F4, (20*S*)-OCH_2_CH_3_-Rg2, and (20*R*)-OCH_2_CH_3_-Rg2, respectively.

#### 2.2.2. Identification of Hydration Adducts

Compounds **2** and **3** exhibit a relative molecular mass of 802.6 and an aglycone ion at *m*/*z* 493.4, both 18.0 Da higher than those of (20*S*/*R*)-Rg2, indicating that they are hydration products of the Rg2 epimers. As shown in the MS^3^ spectra of the ion at *m*/*z* 493.4 (Figures S7B and S8B), product ions at *m*/*z* 475.4, *m*/*z* 417.4, and *m*/*z* 391.3 are observed. The 58.0 Da neutral loss between the ions at *m*/*z* 475.4 and *m*/*z* 417.4 corresponds to the fragment C_3_H_6_O, which is generated by dissociation of a tertiary alcohol with two methyl groups at C-25, formed upon hydration on the C-24(25) double bond. This characteristic neutral loss provides evidence that the hydroxyl group is attached to C-25 in accordance with Markovnikov’s rule. Therefore, compounds **2** and **3** are assigned as (20*S*)-Rf2 and (20*R*)-Rf2, respectively.

The aglycone ion of compounds **1** and **4** exhibits identical spectral features to that of (20*S*/*R*)-Rf2 (Figures S9 and S10), confirming that they possess the same 25-hydroxyl-PPT aglycone. With a relative molecular mass of 656.5, corresponding to the loss of a rhamnosyl substituent from (20*S*/*R*)-Rf2 at the C-6 position, compounds **1** and **4** are identified as (20*S*)-25-OH-Rh1 and (20*R*)-25-OH-Rh1, respectively. Notably, their spectra also exhibit the characteristic 58.0 Da neutral loss associated with C-25 hydroxylation.

Similarly, compounds **5** and **6** exhibit a 58.0 Da neutral loss from the aglycone ion at *m*/*z* 475.4 (Figure S11), which indicates C-25 hydroxylation. However, unlike compounds **2** and **3**, compounds **5** and **6** have the same relative molecular mass of 784.6 and degree of unsaturation as (20*S*/*R*)-Rg2. This suggests that the 18.0 Da mass increase from hydration is counterbalanced by dehydration at C-20, resulting in the formation of Δ20(21) and Δ20(22) derivatives. Therefore, compounds **5** and **6** are assigned as 25-OH-Rg6 and 25-OH-F4, respectively.

#### 2.2.3. Identification of Cyclic Ether Derivatives

**Figure 8:**
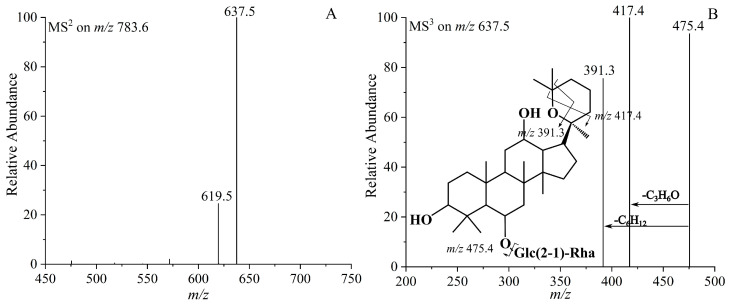
MS^2^ spectrum of the [M−H]^−^ ion at *m*/*z* 783.6 (**A**), fragmentation pathways, and MS^3^ spectrum of the product ion at *m*/*z* 637.5 (**B**) from ginsenoside (20*S*, 25)-epoxy-Rg2.

Compounds **15** and **16** represent another pair of isomers of (20*S*/*R*)-Rg2, both exhibiting a relative molecular mass of 784.6. As shown in Figure 8 and Figure S12, their MS^n^ spectra are largely consistent with those of (20*S*/*R*)-Rg2, except for the presence of a distinctive product ion at *m*/*z* 417.4. Notably, two characteristic neutral losses of 58.0 Da and 84.1 Da are observed from the aglycon ion at *m*/*z* 475.4, corresponding to the fragmentation of the hydroxylated C-25 and the intact olefin chain, respectively. Their coexistence appears contradictory under conventional hydration or dehydration mechanisms. The most plausible explanation is intramolecular cyclization between the C-20 hydroxyl group and the C-24(25) double bond, forming an ether bridge between C-20 and C-25. As illustrated in Figure 8B and Figure S12B, the 58.0 Da loss arises from concurrent cleavage of the C-C and C-O bonds adjacent to C-25, whereas the 84.1 Da loss results from cleavage of the C-20(21) and C-25(O) within the constrained cyclic structure. Therefore, compounds **15** and **16** are assigned as cyclic ether derivatives of (20*S*/*R*)-Rg2, specifically (20*S*, 25)-epoxy-Rg2 and (20*R*, 25)-epoxy-Rg2, respectively. This cyclization preserves the molecular mass but alters the olefin chain connectivity, enabling unique fragmentation patterns.

#### 2.2.4. Identification by Comparison with Authentic Standards

In contrast to the isomeric transformation products discussed above, a distinct set of transformation products was identified by comparison of retention times and MS^n^ spectra with those of authentic standards, revealing alternative reaction pathways under acidic aqueous conditions. Compounds **11**, **12**, **13**, **14**, **19**, **20**, **22**, and **24** are identified as (20*S*)-Rg2, (20*R*)-Rg2, (20*S*)-Rh1, (20*R*)-Rh1, Rg6, F4, Rk3, and Rh4, respectively (Figures S13–S16). These compounds represent sequential deglycosylation and dehydration products derived from ginsenoside Re. Specifically, (20*S*/*R*)-Rg2 arise from cleavage of the C-20 glucosyl substituent of Re, followed by removal of the rhamnose unit at C-6 to yield (20*S*/*R*)-Rh1, both retaining the typical dammarane-type Δ24(25) olefin. Dehydration at the C-20 hydroxyl group of the Rg2 epimers generates Rg6 and F4, featuring C-20(21) and C-20(22) double bonds, respectively. Further deglycosylation at C-6 of these dehydrated intermediates gives rise to Rk3 and Rh4, which possess the minimal number of glycosyl substituents and exhibit a highly rigid triterpenoid core due to the formation of the conjugated diene system.

To validate the structural assignments of the transformation products derived from MS^n^ analysis, HRMS was performed in both all-ion fragmentation and data-dependent MS^2^ modes using normalized collision energy. As summarized in Table 1, the product ion profiles of all 24 compounds showed excellent consistency between HRMS and MS^n^ data. Accurate mass measurements of the key fragment ions confirmed the elemental compositions proposed in the structural identification. This correlation highlights the reliability and precision of the HPLC-MS^n^ approach for structural characterization in this study.

### 2.3. Transformation Pathways and Mechanisms of Ginsenoside Re in Aqueous Ethanol

**Scheme 1:**
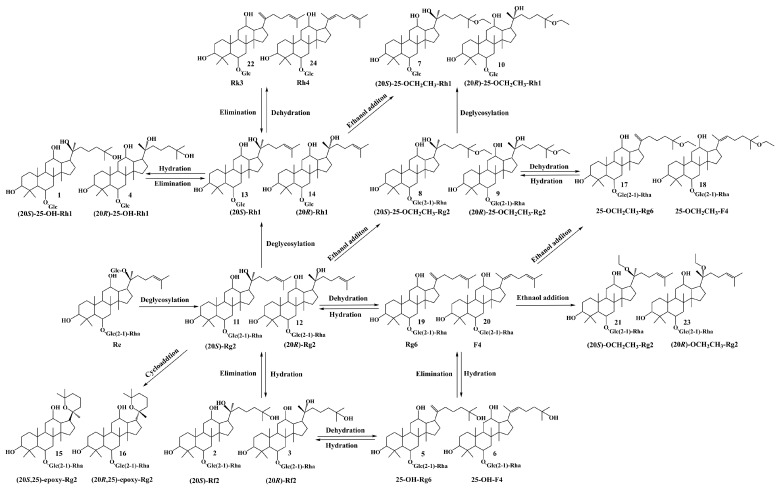
Transformation pathways of ginsenoside Re in aqueous ethanol.

The catalytic transformation of ginsenoside Re over HSiW@mSiO_2_ in aqueous ethanol yielded 24 rare ginsenosides, including seven pairs of isomers, one set of four isomers, and one set of six isomers. As illustrated in Scheme 1, the transformation cascade centers on the formation and subsequent transformation of the key intermediates, (20*S*/*R*)-Rg2, where deglycosylation, elimination, epimerization, nucleophilic addition, and intramolecular cyclization proceed concurrently to generate structural diversity.

#### 2.3.1. Regioselective Deglycosylation

The transformation initiates with the selective hydrolysis of the outer glucosyl substituent at the C-20 position of ginsenoside Re, preferentially over the disaccharide substituent at the C-6 position, yielding (20*S*/*R*)-Rg2 as the primary intermediate. This regioselectivity is rationalized by the carbenium ion mechanism: cleavage of the C-20 glycosidic bond generates a tertiary carbocation, which is thermodynamically more stable than the secondary carbocation formed by C-6 glycosidic bond cleavage due to steric constraints in the aglycone framework [9]. This is further supported by the observation that most transformation products retain the C-6 disaccharide substituent, indicating lower reactivity of the C-6 glycosidic bond under the reaction conditions. Consequently, under HSiW@mSiO_2_-catalyzed acidic conditions, the C-20 glycosidic bond is hydrolyzed more readily, establishing (20*S*/*R*)-Rg2 as the key intermediate for subsequent transformations.

#### 2.3.2. E1 Dehydration

Following the formation of (20*S*/*R*)-Rg2, the hydroxyl group at the C-20 position undergoes E1 elimination under acidic conditions. In this unimolecular process, the rate-determining step is the formation of a carbocation intermediate, whose stability determines the activation barrier and overall reaction rate [9,37]. The kinetics are first-order with respect to the concentration of (20*S*/*R*)-Rg2. In the early reaction stage, the high concentration of (20*S*/*R*)-Rg2 promotes efficient dehydration, leading to the formation of positional isomers Rg6 and F4, which are characterized by C-20(21) and C-20(22) double bonds, respectively. These newly formed double bonds, along with the inherent C-24(25) double bond of the aglycone, serve as critical reactive sites for subsequent nucleophilic addition reactions.

#### 2.3.3. Nucleophilic Addition and Cyclization

Under acidic conditions, the double bonds are protonated to form tertiary carbocation intermediates, which can be trapped by nucleophiles such as water or ethanol. The stability of these intermediates is primarily influenced by hyperconjugation and the inductive effects from the adjacent alkyl groups. These effects determine the regioselectivity of the addition, whereby the functional groups preferentially attach to the C-20 or C-25 positions in accordance with Markovnikov’s rule [38,39,40]. This pathway yields six hydroxylated products and eight ethoxylated products. Notably, while tertiary alcohols can undergo acid-catalyzed dehydration to regenerate the olefins, ethoxyl groups are poor leaving groups under these conditions, rendering ethanol addition effectively irreversible. This kinetic trapping effect leads to the progressive accumulation of ethoxylated products despite the lower nucleophilicity of ethanol. Therefore, the differential reversibility of hydration versus ethanol addition plays a decisive role in product distribution.

In addition to intermolecular addition, the C-20 hydroxyl group in Rg2 can act as an intramolecular nucleophile. Upon protonation, it attacks the electrophilic C-25 of the C-24(25) double bond, yielding the 20,25-epoxy-Rg2 epimers. This intramolecular cyclization competes with intermolecular nucleophilic addition and is influenced by solvent polarity, substrate concentration, and molecular conformation.

In summary, the structural diversity of the 24 rare ginsenosides arises from a combination of acid-catalyzed transformations initiated by selective deglycosylation, with the inherent C-24(25) double bond serving as a key reactive center for both intermolecular nucleophilic addition and intramolecular cyclization. Concurrently, dehydration at C-20 generates transient olefins that further expand the product profile through solvent-dependent trapping. The aqueous ethanol medium thus acts not only as a solvent but also as a co-reactant, enabling the efficient generation of structurally diverse and potentially bioactive ginsenoside derivatives.

### 2.4. Effects of Reaction Conditions on Ginsenoside Re Transformation in Aqueous Ethanol

#### 2.4.1. Time Course Analysis of Reaction Pathway

**Figure 9:**
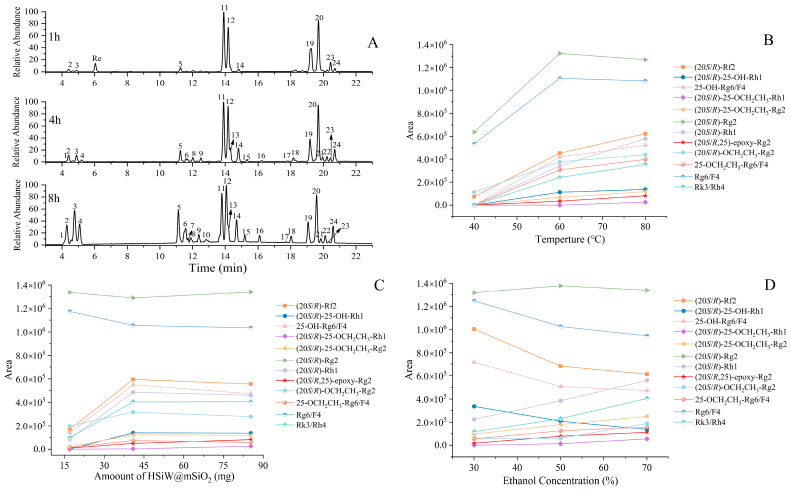
TIC of ginsenoside Re transformation products in aqueous ethanol after 1 h, 4 h, and 8 h (**A**). Peak areas of the transformation products at varied temperature (**B**), catalyst amount (**C**), and ethanol concentration (**D**).

Time-course analysis (Figure 9A) elucidated the sequential nature of the transformation pathways. Within the 1 h of reaction in 70% ethanol, the primary products of (20*S*/*R*)-Rg2, Rg6, and F4 emerged. The high abundance of the (20*S*/*R*)-Rg2 is indicative of their role as predominant products. The concurrent formation of Rg6 and F4 indicates that E1 elimination at C-20 proceeds efficiently under mild acidic conditions, thus surpassing competing addition pathways. By 4 h, complete consumption of Re is observed, and the full spectrum of 24 transformation products has emerged. The extension of the reaction time to 8 h resulted in a substantial decrease in the peak areas of early-stage intermediates, accompanied by a notable increase in the abundance of subsequent products. This shift reflects the consumption of primary intermediates in subsequent reactions, including nucleophilic additions and further deglycosylation at the C-6 position, highlighting the dynamic and multi-stage character of the overall transformation.

#### 2.4.2. Temperature-Dependent Reaction Kinetics

The reaction temperature was found to have a significant effect on both the rate and selectivity of product formation. At 40 °C, the reaction proceeded selectively, yielding predominantly Rg2 epimers, their ethoxylated products (20*S*/*R*)-OCH_2_CH_3_-Rg2, and the dehydration products Rg6 and F4 after 8 h (Figure 9B). This demonstrates that the primary transformations of deglycosylation, elimination, and initial addition are accessible even under mild thermal conditions, thereby indicating the high catalytic efficiency of HSiW@mSiO_2_. Elevating the temperature to 80 °C dramatically accelerated the reaction kinetics, enabling the rapid generation of the additional 18 products. Notably, the relative abundance of early intermediates decreased, while advanced transformation products, particularly those derived from nucleophilic addition and C-6 deglycosylation, increased substantially. This temperature-driven shift confirms that higher thermal energy promotes the progression of secondary and tertiary reactions, thereby expanding the structural diversity of the resulting rare ginsenosides.

#### 2.4.3. Catalyst Dosage and Product Distribution

The concentration of accessible Brønsted acid sites, controlled by HSiW@mSiO_2_ dosage, directly modulates reaction efficiency. Increasing the catalyst amount from 17.1 mg to 40.9 mg significantly enhanced the rate of nucleophilic addition of water and ethanol to olefin intermediates (Figure 9C). This observation indicates that an elevated proton availability accelerates key transformation steps such as double bond protonation and carbocation formation. However, further increasing the loading to 85.3 mg resulted in only minimal additional improvements in product yield or distribution. This plateau effect suggests that the system reaches a state of acid site saturation, where the rate of proton transfer is no longer limited by catalyst concentration but likely by diffusion or intrinsic kinetic barriers, indicating optimal catalytic efficiency at moderate dosages.

#### 2.4.4. Solvent-Dependent Nucleophilic Addition Selectivity

The ethanol/water ratio emerged as a critical parameter for modulating reaction selectivity. At 30% ethanol, hydration products, such as Rf2 epimers, 25-OH-Rg6, and 25-OH-F4, predominated, indicating that water acts as the dominant nucleophile under aqueous-rich conditions (Figure 9D). In contrast, an increase in the ethanol concentration to 70% led to a substantial reversal in selectivity, with hydration products decreasing considerably and ethoxylated products becoming significantly more abundant. This clear trend demonstrates a competitive nucleophilic addition mechanism between water and ethanol at electrophilic carbons generated upon protonation of C-20(21), C-20(22), and C-24(25) double bonds. Thus, solvent composition acts as an effective tool to modulate product distribution, with elevated ethanol concentrations enabling access to novel ethoxylated rare ginsenosides.

### 2.5. Reusability and Structural Stability of HSiW@mSiO_2_ in Sequential Cycles

**Figure 10:**
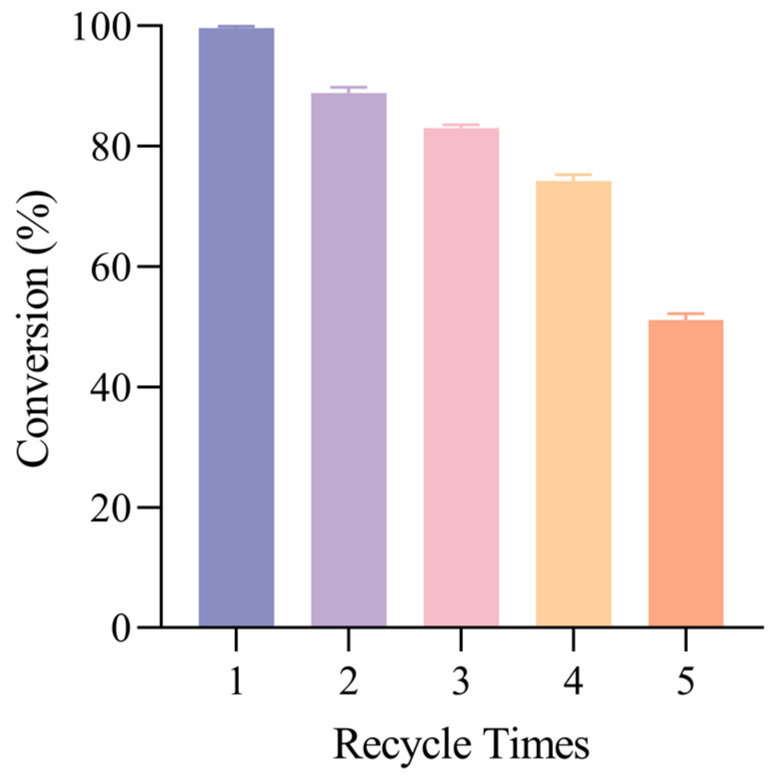
Transformation rate of ginsenoside Re catalyzed by HSiW@mSiO_2_ at different cycle times.

The heterogeneous catalyst HSiW@mSiO_2_ can be readily separated from the reaction mixture by centrifugation after promoting the transformation of ginsenoside Re in 70% aqueous ethanol solution at 80 °C for 4 h, enabling its reuse across consecutive runs. After isolation, the catalyst was washed, dried under vacuum at 50 °C, and directly used in the next cycle. As shown in Figure 10, full substrate transformation was maintained during the first two cycles, but a gradual decrease in activity became evident thereafter, reaching about 50% conversion by the fifth cycle. This deactivation is primarily attributed to partial leaching of the active HSiW species from the mesoporous silica framework, as supported by the ICP-MS analysis showing a decrease in tungsten content from 105.6 mg/g in the fresh catalyst to 72.3 mg/g after five cycles, alongside subtle degradation of the framework under prolonged acidic conditions. Together, these factors reduce the density of accessible Brønsted acid sites. These results underscore the critical role of immobilization stability in determining long-term catalyst performance.

## 3. Materials and Methods

### 3.1. Chemicals and Materials

12-tungstosilicic acid (H_4_SiW_12_O_40_) of analytical grade and authentic ginsenoside standards (purity ≥ 98%) of Re, (20*S*)-Rg2, (20*R*)-Rg2, (20*S*)-Rh1, (20*R*)-Rh1, Rh4, Rk3, Rg6, and F4, were purchased from Shanghai Yuanye Biological Technology Co., Ltd. (Shanghai, China). Tetraethyl orthosilicate (TEOS), hexadecyl trimethyl ammonium bromide (CTAB), and ammonium hydroxide (NH_3_·H_2_O) of analytical grade were purchased from Shanghai Macklin Biochemical Co., Ltd. (Shanghai, China). HPLC-grade acetonitrile, ethanol, and formic acid were purchased from Tedia (Fairfield, OH, USA) and Thermo Fisher Scientific (Waltham, MA, USA), respectively. Ultrapure water was prepared using a Milli-Q water purification system (Merck Millipore, Burlington, MA, USA).

### 3.2. Instruments and Conditions

XRD patterns were recorded using a TDM-10 diffractometer (Dandong Tongda Technology Co., Ltd., Dandong, China), which was equipped with Cu Kα radiation (λ = 1.5418 Å). Low-angle XRD data (1–5° 2θ) were collected to investigate the mesoporous structure, and wide-angle XRD data (10–70° 2θ) were acquired to assess crystallinity. Measurements were performed at a tube voltage of 40 kV and a current of 30 mA. FTIR spectra were recorded on a Thermo Scientific Nicolet iS10 spectrometer (Waltham, MA, USA) in the wavenumber range of 4000–400 cm^−1^ with a resolution of 4 cm^−1^. Samples were prepared as KBr pellets prior to analysis. TEM images were acquired using a JEOL JEM-2100F electron microscope (JEOL Ltd., Tokyo, Japan) operated at an accelerating voltage of 200 kV. Samples were dispersed in ethanol, sonicated, and drop-cast onto carbon-coated copper grids prior to analysis. Inductively coupled plasma mass spectrometry (ICP-MS) was performed on an Agilent 7800 instrument (Agilent Technologies, Santa Clara, CA, USA) to determine the elemental content.

HPLC analysis was carried out on a Thermo Scientific Ultimate 3000 system (San Jose, CA, USA) equipped with a Syncronis C18 column (100 × 2.1 mm, 1.7 μm). The column temperature was maintained at 35 °C. The mobile phase consisted of phase A (water containing 0.1% *v*/*v* formic acid) and phase B (acetonitrile). The flow rate was set to 0.2 mL/min. The gradient elution program was set as follows: 0–5 min (25% B), 5–8 min (30–36% B), 8–15 min (36–48% B), 15–20 min (48–70% B), 20–25 min (70–90% B), 25–28 min (90% B), and 28–34 min (90–25% B). The injection volume was 2.0 μL.

HRMS analysis was performed on a Q-Exactive hybrid quadrupole-Orbitrap mass spectrometer (Thermo Fisher Scientific, San Jose, CA, USA), while MS^n^ experiments were conducted on an LTQ XL ion trap mass spectrometer (Thermo Fisher Scientific, San Jose, CA, USA). Both instruments were coupled online with the HPLC system via electrospray ionization (ESI) sources operated in negative-ion mode, which provided enhanced ionization efficiency for ginsenosides. The ion source parameters were optimized as follows: sheath gas flow rate, 35 (arbitrary units); auxiliary gas flow rate, 10 (arbitrary units); sweep gas flow rate, 1 (arbitrary unit); capillary temperature, 320 °C; and capillary voltage, −3.2 kV. The full-scan mass range was set to *m*/*z* 100–2000 with a resolution of 70,000 (at *m*/*z* 200), while the product ion scan range was set to *m*/*z* 200–1000.

### 3.3. Sample Preparation

#### 3.3.1. Preparation of mSiO_2_

1.76 g of CTAB was dissolved in 561 mL of ultrapure water. 3.20 mL of NH_3_·H_2_O was then added, and the mixture was stirred for 30 min at room temperature. Subsequently, 9.33 mL of TEOS was added dropwise under vigorous stirring, and the reaction mixture was further stirred for 2 h. The mixture was aged at room temperature for 20 h. Following aging, the resulting gel products were collected by vacuum filtration using a Büchner funnel and filter paper, and washed repeatedly with a 50% ethanol-water solution (*v*/*v*) to remove CTAB and residual reactants. The collected solid was dried overnight at 80 °C, followed by calcination in air at 550 °C for 4 h. The final product was stored under vacuum.

#### 3.3.2. Preparation of HSiW@mSiO_2_

0.6 g of HSiW was dissolved in 60 mL of ultrapure water, followed by the addition of 2 g of the as-prepared mSiO_2_. The resulting mixture was stirred at room temperature for 22 h, and the mixture was then transferred to a water bath at 50 °C to allow slow evaporation of the solvent. The resulting solid was dried at 100 °C for 12 h, followed by calcination in air at 300 °C for 2 h [10].

#### 3.3.3. Transformation of Ginsenoside Re

2.0 mg of ginsenoside Re authentic standard was dissolved in 2 mL of 70% (*v*/*v*) ethanol aqueous solution. Then, 37.8 mg of the HSiW@mSiO_2_ catalyst was added to the solution. The reaction mixture was immediately transferred to a temperature-controlled shaking water bath set at 80 °C and reacted for 8 h with continuous shaking. After the reaction, the mixture was cooled to room temperature and then centrifuged at 3000 rpm for 2 min to pellet the catalyst. A 200 μL aliquot of the supernatant was diluted to 1 mL with ethanol, then passed through a 0.22 μm syringe filter prior to HPLC-MS analysis. The catalyst precipitate was collected, washed with 50% (*v*/*v*) ethanol aqueous solution, and dried under vacuum at 50 °C. The recovered catalyst was reused in consecutive transformation cycles of ginsenoside Re.

## 4. Conclusions

In this study, a mesoporous silica-supported heteropolyacid catalyst HSiW@mSiO_2_ was developed for the transformation of ginsenoside Re in aqueous ethanol solution. The catalyst promoted a cascade of acid-mediated reactions, including deglycosylation, epimerization, dehydration, nucleophilic addition, and intramolecular cyclization yielding 24 structurally diverse rare ginsenosides, as characterized by HPLC-MS^n^/HRMS. These products include seven pairs of isomers, one set of four isomers and one set of six isomers mainly arising from C-20 epimerization or differences in double bond location. Notably, eight ethoxylated PPT-type ginsenosides were formed via ethanol trapping of reactive carbocation intermediates, including (20*S*/*R*)-OCH_2_CH_3_-Rg2, (20*S*/*R*)-25-OCH_2_CH_3_-Rg2, their derhamnosylated derivatives (20*S*/*R*)-25-OCH_2_CH_3_-Rh1, and dehydrated derivatives 25-OCH_2_CH_3_-Rg6/F4. These ethoxylated products represent novel ginsenoside scaffolds featuring either C-20 or C-25 ethoxyl substitution and highlight the potential of solvent as a nucleophile in generating structurally unique and naturally rare ginsenoside analogs. The reaction efficiency was influenced by solvent composition, temperature, and catalyst loading. Furthermore, HSiW@mSiO_2_ could be readily recovered and reused for multiple cycles, although gradual activity loss was observed. This work demonstrates the utility of solid acid catalysts in enabling selective, tunable, and solvent-directed structural modification of complex natural products, offering a sustainable strategy for expanding the chemical diversity of bioactive ginsenosides.

## Figures and Tables

**Table 1:** HRMS-derived molecular and ion data for ginsenoside Re transformation products in aqueous ethanol.

Peak	Identification	Relative Molecular Mass	Molecular Formula	Measured [M−H]^−^ (*m*/*z*)	Fragment Ions
1	(20*S*)-25-OH-Rh1	656.4494	C_36_H_64_O_10_	655.4478	493.4664 [M−Glc−H]^−^, 475.4215 [M−Glc−H_2_O−H]^−^, 417.4839 [M−Glc−C_3_H_8_O_2_−H]^−^, 391.3672 [M−Glc−C_6_H_14_O−H]^−^
2	(20*S*)-Rf2	802.5073	C_42_H_74_O_14_	801.5087	655.5321 [M−Rha−H]^−^, 637.5381 [M−Rha−H_2_O−H]^−^, 493.4871 [M−Rha−Glc−H]^−^, 475.4267 [M−Glc−Rha−H_2_O−H]^−^, 417.4835 [M−Rha−Glc–C_3_H_8_O_2_−H]^−^, 391.3662 [M−Glc−Rha−C_6_H_14_O]^−^
3	(20*R*)-Rf2	802.5073	C_42_H_74_O_14_	801.5081	655.5329 [M−Rha−H]^−^, 637.5385 [M−Rha−H_2_O−H]^−^, 493.4873 [M−Rha−Glc−H]^−^, 475.4361 [M−Glc−Rha−H_2_O−H]^−^, 417.4830 [M−Rha−Glc−C_3_H_8_O_2_−H]^−^, 391.3669 [M−Glc−Rha−C_6_H_14_O]^−^
4	(20*R*)-25-OH-Rh1	656.4494	C_36_H_64_O_10_	655.4481	493.4668 [M−Glc−H]^−^, 475.4210 [M−Glc−H_2_O−H]^−^, 417.4830 [M−Glc−C_3_H_8_O_2_−H]^−^, 391.3678 [M−Glc−C_6_H_14_O−H]^−^
5	25-OH-Rg6	784.4967	C_42_H_72_O_13_	783.4977	637.5122 [M−Rha−H]^−^, 619.5321 [M−Rha−H_2_O−H]^−^, 475.4131 [M−Glc−Rha−H]^−^, 417.4831 [M−Glc−Rha−C_3_H_6_O]^−^
6	25-OH-F4	784.4967	C_42_H_72_O_13_	783.4982	637.5121 [M−Rha−H]^−^, 619.5322 [M−Rha−H_2_O−H]^−^, 475.4132 [M−Glc−Rha−H]^−^, 417.4832 [M−Glc−Rha−C_3_H_6_O]^−^
7	(20*S*)-25-OCH_2_CH_3_-Rh1	684.4807	C_38_H_68_O_10_	683.4817	521.4324 [M−Glc−H]^−^, 475.4261 [M−Glc−CH_3_CH_2_OH−H]^−^, 457.4261 [M−Glc−CH_3_CH_2_OH−H_2_O−H]^−^, 391.4665 [M−Glc−C_8_H_18_O−H]^−^
8	(20*S*)-25-OCH_2_CH_3_-Rg2	830.5386	C_44_H_78_O_14_	829.5376	683.5325 [M−Rha−H]^−^, 665.5180 [M−Rha−H_2_O−H]^−^, 521.4292 [M−Glc−Rha−H]^−^, 475.4261 [M−Glc−Rha−CH_3_CH_2_OH−H]^−^, 457.4312 [M−Glc−Rha−CH_3_CH_2_OH−H_2_O−H]^−^, 391.3662 [M−Glc−Rha−C_8_H_18_O−H]^−^
9	(20*R*)-25-OCH_2_CH_3_-Rg2	830.5386	C_44_H_78_O_14_	829.5369	683.5323 [M−Rha−H]^−^, 665.5181 [M−Rha−H_2_O−H]^−^, 521.4290 [M−Glc−Rha−H]^−^, 475.4263 [M−Glc−Rha−CH_3_CH_2_OH−H]^−^, 457.4314 [M−Glc−Rha−CH_3_CH_2_OH−H_2_O−H]^−^, 391.3661 [M−Glc−Rha−C_8_H_18_O−H]^−^
10	(20*R*)-25-OCH_2_CH_3_-Rh1	684.4807	C_38_H_68_O_10_	683.4816	521.4326 [M−Glc−H]^−^, 475.4263 [M−Glc−CH_3_CH_2_OH−H]^−^, 457.4262 [M−Glc−CH_3_CH_2_OH−H_2_O−H]^−^, 391.4661 [M−Glc−C_8_H_18_O−H]^−^
11	(20*S*)-Rg2	784.4967	C_42_H_72_O_13_	783.4974	637.5124 [M−Rha−H]^−^, 619.5324 [M−Rha−H_2_O−H]^−^, 475.4133 [M−Glc−Rha−H]^−^, 391.3661 [M−Glc−Rha−C_6_H_12_−H]^−^
12	(20*R*)-Rg2	784.4967	C_42_H_72_O_13_	783.4952	637.5121 [M−Rha−H]^−^, 619.5320 [M−Rha−H_2_O−H]^−^, 475.4134 [M−Glc−Rha−H]^−^, 391.3663 [M−Glc−Rha−C_6_H_12_−H]^−^
13	(20*S*)-Rh1	638.4388	C_36_H_62_O_9_	637.4379	475.4260 [M−Glc−H]^−^
14	(20*R*)-Rh1	638.4388	C_36_H_62_O_9_	637.4385	475.4261 [M−Glc−H]^−^
15	(20*S*, 25)-epoxy-Rg2	784.4967	C_42_H_72_O_13_	784.4958	637.6121 [M−Rha−H]^−^, 619.6324 [M−Rha−H_2_O−H]^−^, 475.5131 [M−Glc−Rha−H]^−^, 417.5831 [M−Glc−Rha−C_3_H_6_O−H]^−^, 391.4 [M−Glc−Rha−C_6_H_12_−H]^−^
16	(20*R*, 25)-epoxy-Rg2	784.4967	C_42_H_72_O_13_	783.4964	637.6121 [M−Rha−H]^−^, 619.6324 [M−Rha−H_2_O−H]^−^, 475.5131 [M−Glc−Rha−H]^−^, 417.5831 [M−Glc−Rha−C_3_H_6_O−H]^−^, 391.4 [M−Glc−Rha−C_6_H_12_−H]^−^
17	25-OCH_2_CH_3_-Rg6	812.5280	C_44_H_76_O_13_	811.5273	665.5441 [M−Rha−H]^−^, 647.5322 [M−Rha−H_2_O−H]^−^, 503.4433 [M−Glc−Rha−H]^−^, 457.4133 [M−Glc−Rha−CH_3_CH_2_OH−H]^−^
18	25-OCH_2_CH_3_-F4	812.5280	C_44_H_76_O_13_	811.5279	665.5443 [M−Rha−H]^−^, 647.5321 [M−Rha−H_2_O−H]^−^, 503.4431 [M−Glc−Rha−H]^−^, 457.4131 [M−Glc−Rha−CH_3_CH_2_OH−H]^−^
19	Rg6	766.4862	C_42_H_70_O_12_	765.4842	619.5321 [M−Rha−H]^−^, 601.5550 [M−Rha−H_2_O−H]^−^, 457.4131 [M−Glc−Rha−H]^−^
20	F4	766.4862	C_42_H_70_O_12_	765.4849	619.5324 [M−Rha−H]^−^, 601.5551 [M−Rha−H_2_O−H]^−^, 457.4133 [M−Glc−Rha−H]^−^
21	(20*S*)-OCH_2_CH_3_-Rg2	812.5280	C_44_H_76_O_13_	811.5266	665.5443 [M−Rha−H]^−^, 647.5324 [M−Rha−H_2_O−H]^−^, 503.4432 [M−Glc−Rha−H]^−^, 457.4136 [M−Glc−Rha−CH_3_CH_2_OH−H]^−^
22	Rk3	620.4283	C_36_H_60_O_8_	619.4269	457.4138 [M−Glc−H]^−^
23	(20*R*)-OCH_2_CH_3_-Rg2	812.5280	C_44_H_76_O_13_	811.5265	665.5442 [M−Rha−H]^−^, 647.5321 [M−Rha−H_2_O−H]^−^, 503.4430 [M−Glc−Rha−H]^−^, 457.4134 [M−Glc−Rha−CH_3_CH_2_OH−H]^−^
24	Rh4	620.4283	C_36_H_60_O_8_	619.4274	457.4133 [M−Glc−H]^−^

## Data Availability

The data supporting this article have been included as part of the Supplementary Materials.

## Supplementary Materials

The following supporting information can be downloaded at: https://www.mdpi.com/article/10.3390/molecules30244753/s1, Figure S1: FTIR spectra of HSiW, mSiO_2_, and HSiW@mSiO_2_; Figure S2: The total ion chromatogram of untreated ginsenoside Re. Figure S3: MS^2^ spectrum of the [M−H]^−^ ion at *m*/*z* 829.6 (A), fragmentation pathways, and MS^3^ spectrum of the product ion at *m*/*z* 521.4 (B) from ginsenoside (20*R*)-25-OCH_2_CH_3_-Rg2; Figure S4: MS^2^ spectrum of the [M−H]^−^ ion at *m*/*z* 683.5 (A), fragmentation pathways, and MS^3^ spectrum of the product ion at *m*/*z* 521.4 (B) from ginsenoside (20*R*)-25-OCH_2_CH_3_-Rh1; Figure S5: MS^2^ spectrum of the [M−H]^−^ ion at *m*/*z* 811.6 from ginsenoside 25-OCH_2_CH_3_-F4; Figure S6: MS^2^ spectra of the [M−H]^−^ ion at *m*/*z* 811.6 from ginsenosides (20*S*)-OCH_2_CH_3_-Rg2 (A) and (20*R*)-OCH_2_CH_3_-Rg2 (B); Figure S7: MS^2^ spectrum of the [M−H]^−^ ion at *m*/*z* 801.6 (A), fragmentation pathways, and MS^3^ spectrum of the product ion at *m*/*z* 493.4 (B) from ginsenoside (20*S*)-Rf2; Figure S8: MS^2^ spectrum of the [M−H]^−^ ion at *m*/*z* 801.6 (A), fragmentation pathways, and MS^3^ spectrum of the product ion at *m*/*z* 493.4 (B) from ginsenoside(20*R*)-Rf2; Figure S9: MS^2^ spectrum of the [M−H]^−^ ion at *m*/*z* 655.5 (A), fragmentation pathways, and MS^3^ spectrum of the product ion at *m*/*z* 493.4 (B) from ginsenoside (20*S*)-25-OH-Rh1; Figure S10: MS^2^ spectrum of the [M−H]^−^ ion at *m*/*z* 655.5 (A), fragmentation pathways, and MS^3^ spectrum of the product ion at *m*/*z* 493.4 (B) from ginsenoside (20*R*)-25-OH-Rh1; Figure S11: MS^2^ spectra of the [M−H]^−^ ion at *m*/*z* 783.6 from ginsenosides 25-OH-Rg6 (A) and 25-OH-F4 (B); Figure S12: MS^2^ spectrum of the [M−H]^−^ ion at *m*/*z* 783.6 (A), fragmentation pathways, and MS^3^ spectrum of the product ion at *m*/*z* 637.5 (B) from ginsenoside (20*R*,25)-epoxy-Rg2; Figure S13: MS^2^ spectra of the [M−H]^−^ ion at *m*/*z* 783.6 from ginsenosides (20*S*)-Rg2 (A) and (20*R*)-Rg2 (B); Figure S14: MS^2^ spectra of the [M−H]^−^ ion at *m*/*z* 637.5 from ginsenosides (20*S*)-Rh1 (A) and (20*R*)-Rh1 (B); Figure S15: MS^2^ spectra of the [M−H]^−^ ion at *m*/*z* 765.6 from ginsenosides Rg6 (A) and F4 (B); Figure S16: MS^2^ spectra of the [M−H]^−^ ion at *m*/*z* 619.5 from ginsenosides Rk3 (A) and Rh4 (B).
