# Supplementary material for: Catalytic Transformation of Ginsenoside Re over Mesoporous Silica-Supported Heteropoly Acids: Generation of Diverse Rare Ginsenosides in Aqueous Ethanol Revealed by HPLC-HRMSn
Source: Molecules. 2025 Dec 12;30(24):4753. doi: 10.3390/molecules30244753 (PMC12736098; doi:10.3390/molecules30244753)
Supplement: Supplementary file 1 [file molecules-30-04753-s001.zip › molecules-4011804-supplementary.pdf]

# Supplementary materials

# Catalytic Transformation of Ginsenoside Re over Mesoporous Silica-Supported Heteropoly Acids: Generation of Diverse Rare Ginsenosides in Aqueous Ethanol Revealed by HPLC-HRMS$^n$

**Qi Wang, Yanyan Chang, Bing Li, Zhenxuan Zhang, Mengya Zhao, Huanxi Zhao*, and Yang Xiu***

Jilin Ginseng Academy, Changchun University of Chinese Medicine, Changchun 130117, China;

13278542346@163.com (Q.W.); cyy10141825@163.com (Y.C.); 17614370678@163.com (B.L.); 19969501607@163.com (Z.Z); zmy15981530161@163.com (M.Z.);

* Correspondence: phoenix8713@sina.com (H.Z.); xiuyang@ccucm.edu.cn (Y.X.)

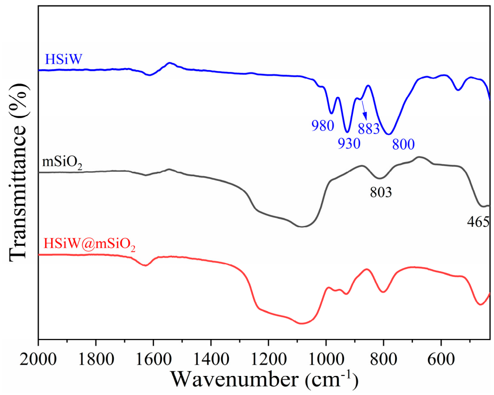


**Figure S1.** FTIR spectra of HSiW, $mSiO_2$, and $HSiW@mSiO_2$.

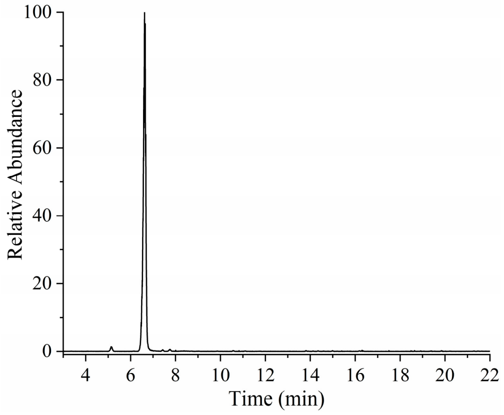


**Figure S2.** The total ion chromatogram of untreated ginsenoside Re.

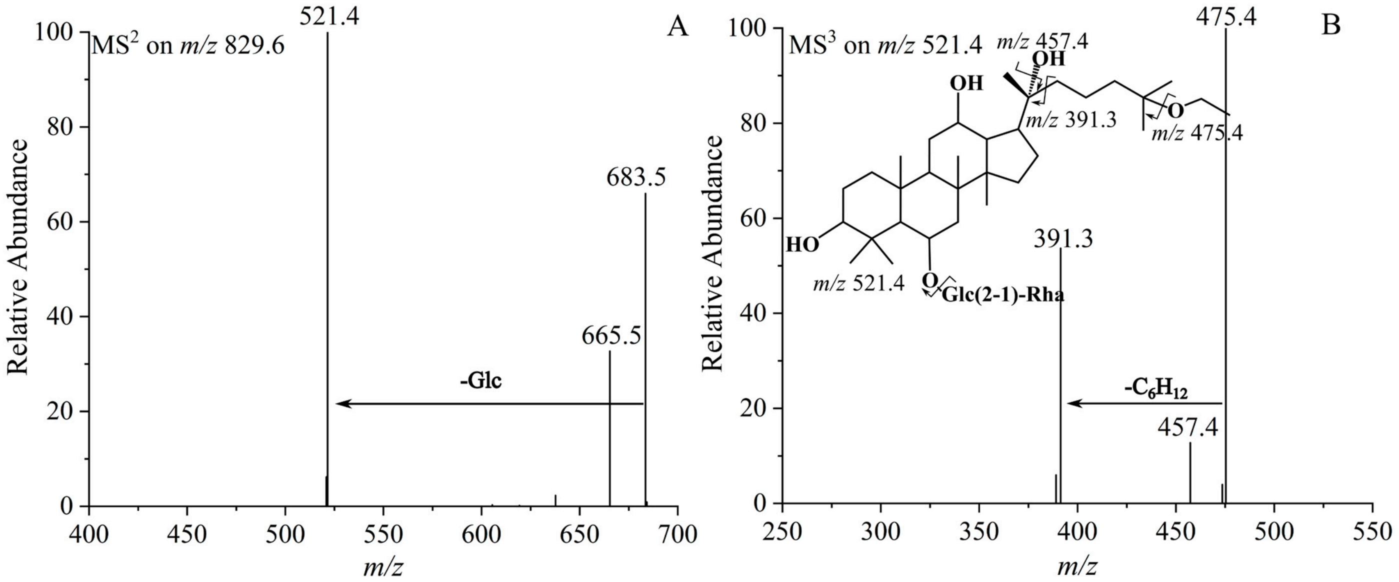


**Figure S3.** $MS^2$ spectrum of the $[M{-}H]^-$ ion at *m/z* 829.6 (A), fragmentation pathways, and $MS^3$ spectrum of the product ion at *m/z* 521.4 (B) from ginsenoside (20*R*)-25-$OCH_2CH_3$-Rg2.

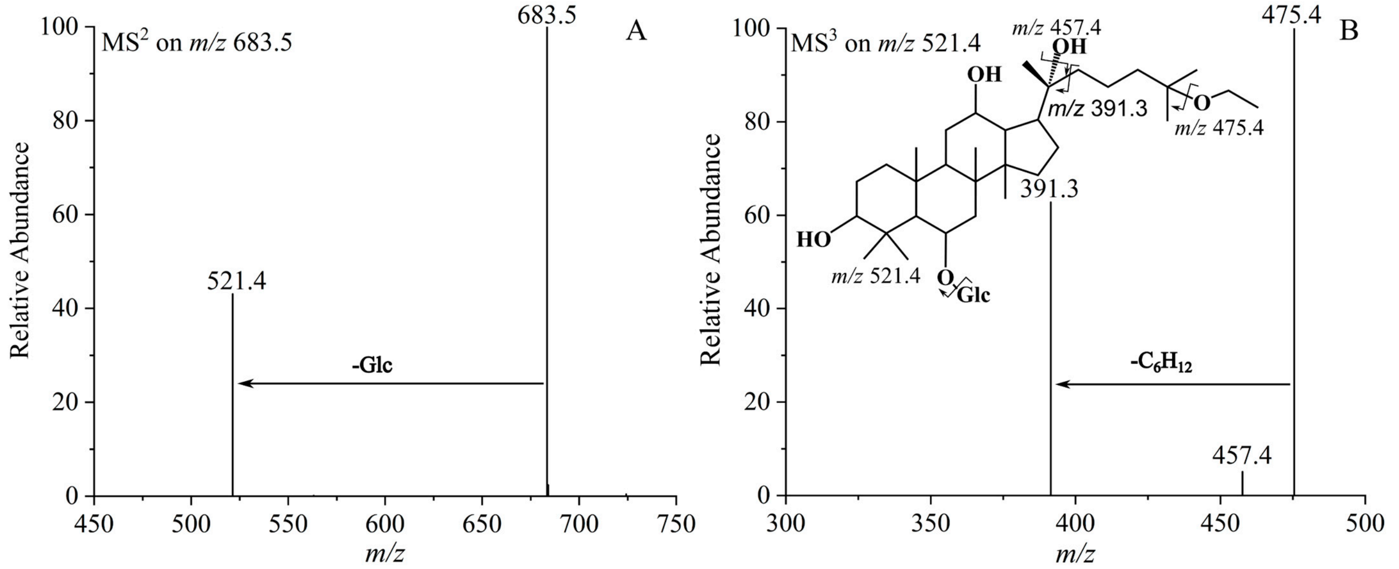


**Figure S4.** $MS^2$ spectrum of the $[M{-}H]^-$ ion at *m/z* 683.5 (A), fragmentation pathways, and $MS^3$ spectrum of the product ion at *m/z* 521.4 (B) from ginsenoside (20*R*)-25-$OCH_2CH_3$-Rh1.

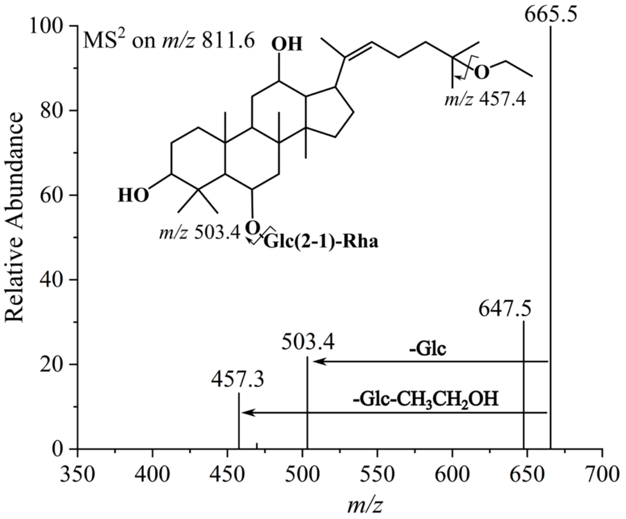


**Figure S5.** MS$^2$ spectrum of the $[M-H]^-$ ion at *m/z* 811.6 from ginsenoside 25-OCH$_2$CH$_3$-F4.

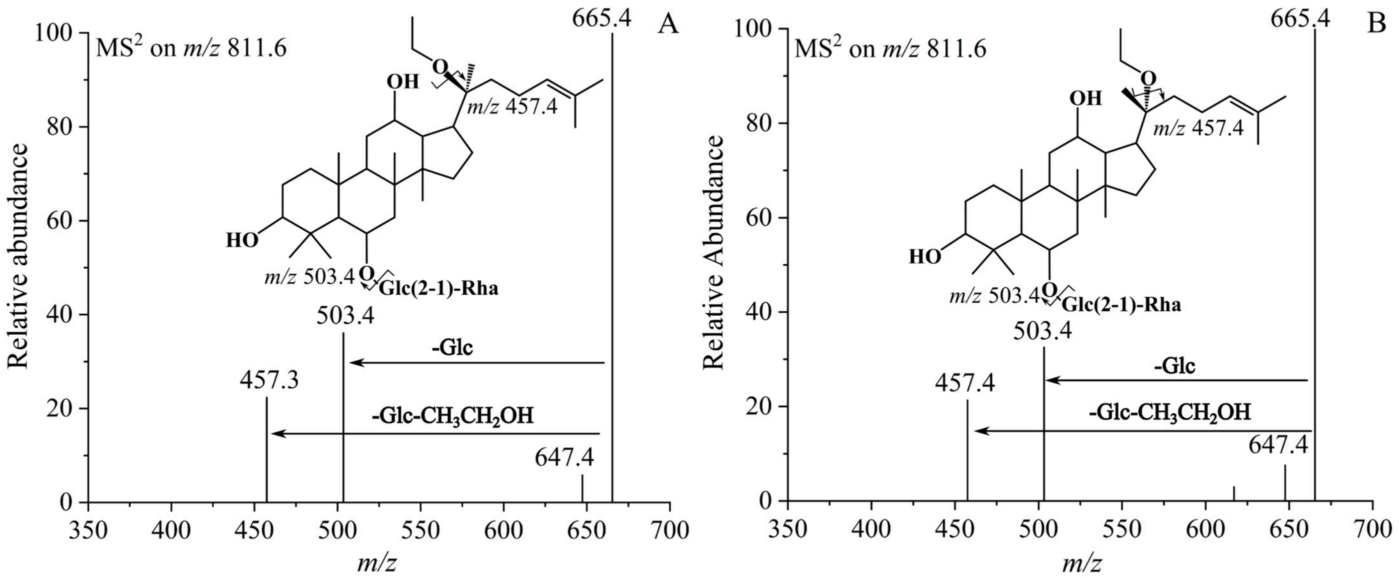


**Figure S6.** MS$^2$ spectra of the $[M-H]^-$ ion at *m/z* 811.6 from ginsenosides (20*S*)-OCH$_2$CH$_3$-Rg2 (A) and (20*R*)-OCH$_2$CH$_3$-Rg2 (B).

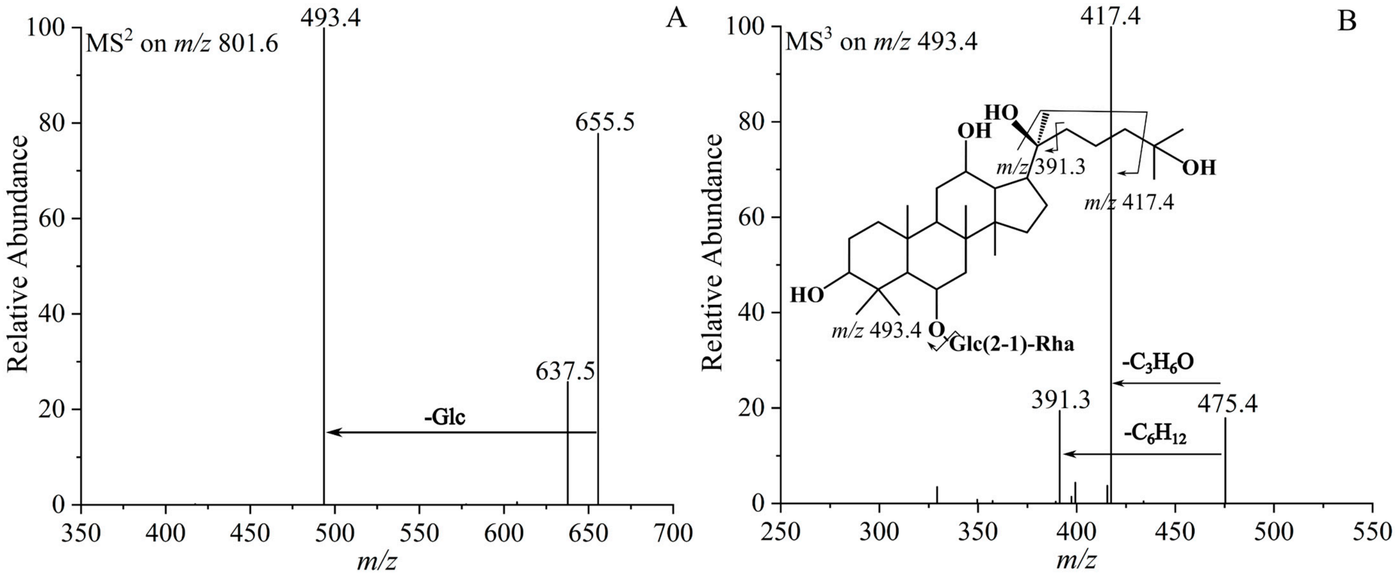


**Figure S7.** $MS^2$ spectrum of the $[M{-}H]^-$ ion at *m/z* 801.6 (A), fragmentation pathways, and $MS^3$ spectrum of the product ion at *m/z* 493.4 (B) from ginsenoside (20*S*)-Rf2.

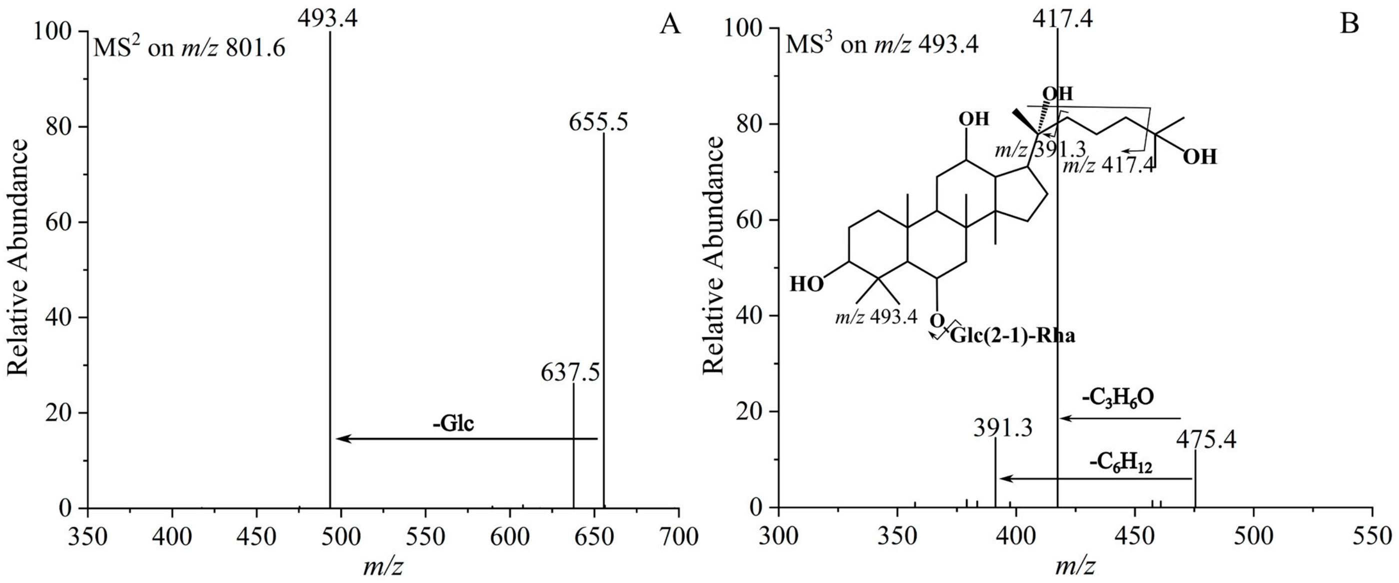


**Figure S8.** $MS^2$ spectrum of the $[M{-}H]^-$ ion at *m/z* 801.6 (A), fragmentation pathways, and $MS^3$ spectrum of the product ion at *m/z* 493.4 (B) from ginsenoside (20*R*)-Rf2.

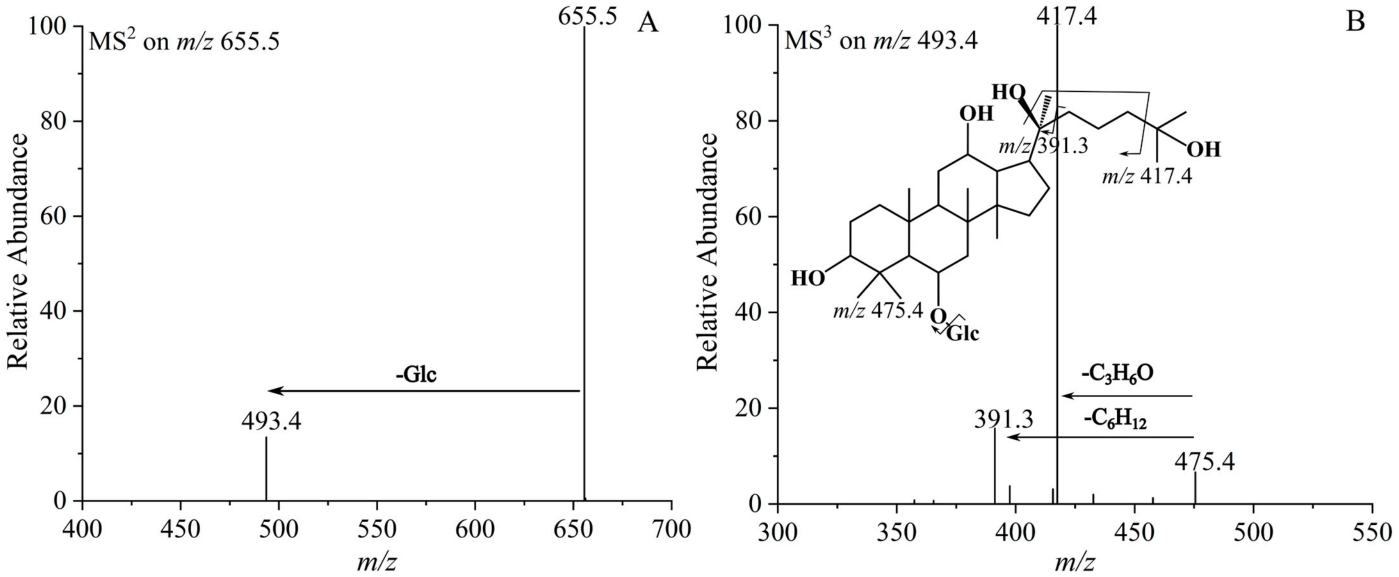


**Figure S9.** MS[2] spectrum of the [M−H][-] ion at $m/z$ 655.5 (A), fragmentation pathways, and MS[3] spectrum of the product ion at $m/z$ 493.4 (B) from ginsenoside (20*S*)-25-OH-Rh1.

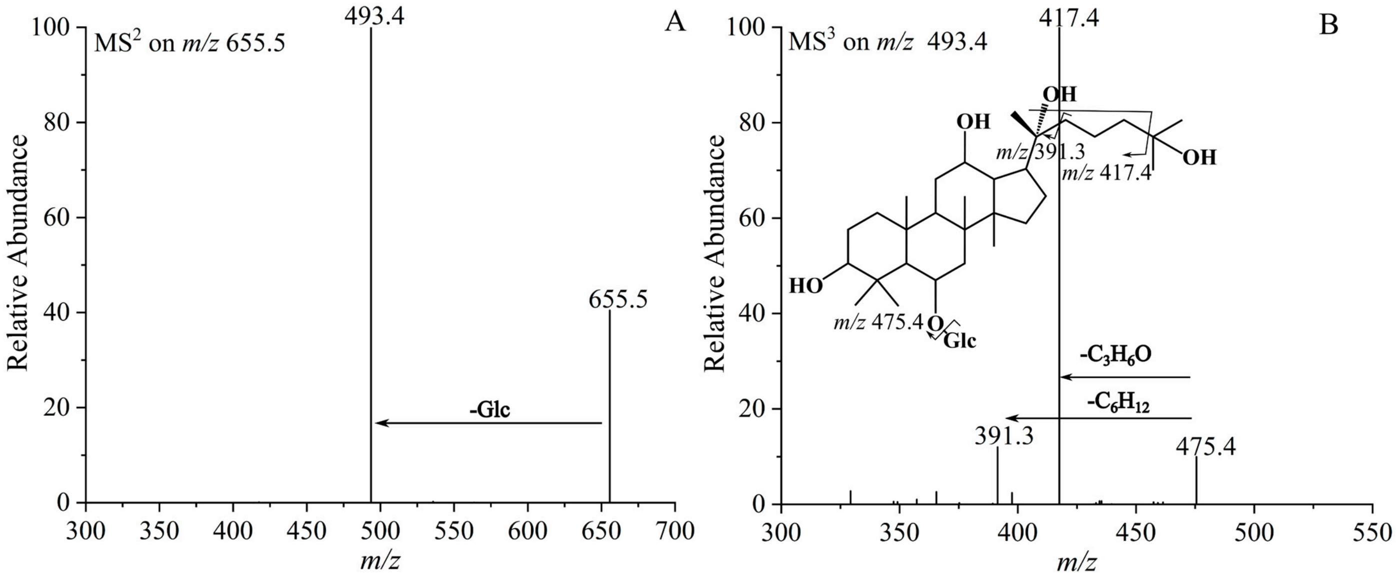


**Figure S10.** MS[2] spectrum of the [M−H][-] ion at $m/z$ 655.5 (A), fragmentation pathways, and MS[3] spectrum of the product ion at $m/z$ 493.4 (B) from ginsenoside (20*R*)-25-OH-Rh1.

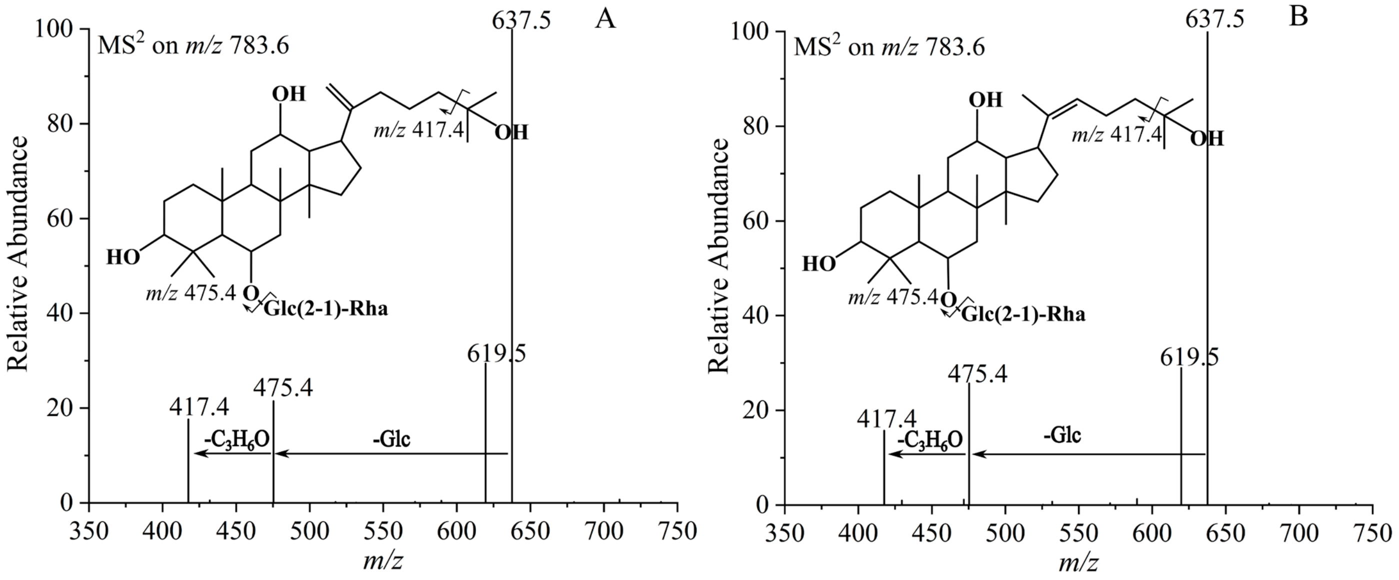


**Figure S11.** MS$^2$ spectra of the $[M{-}H]^-$ ion at $m/z$ 783.6 from ginsenosides 25-OH-Rg6 (A) and 25-OH-F4 (B).

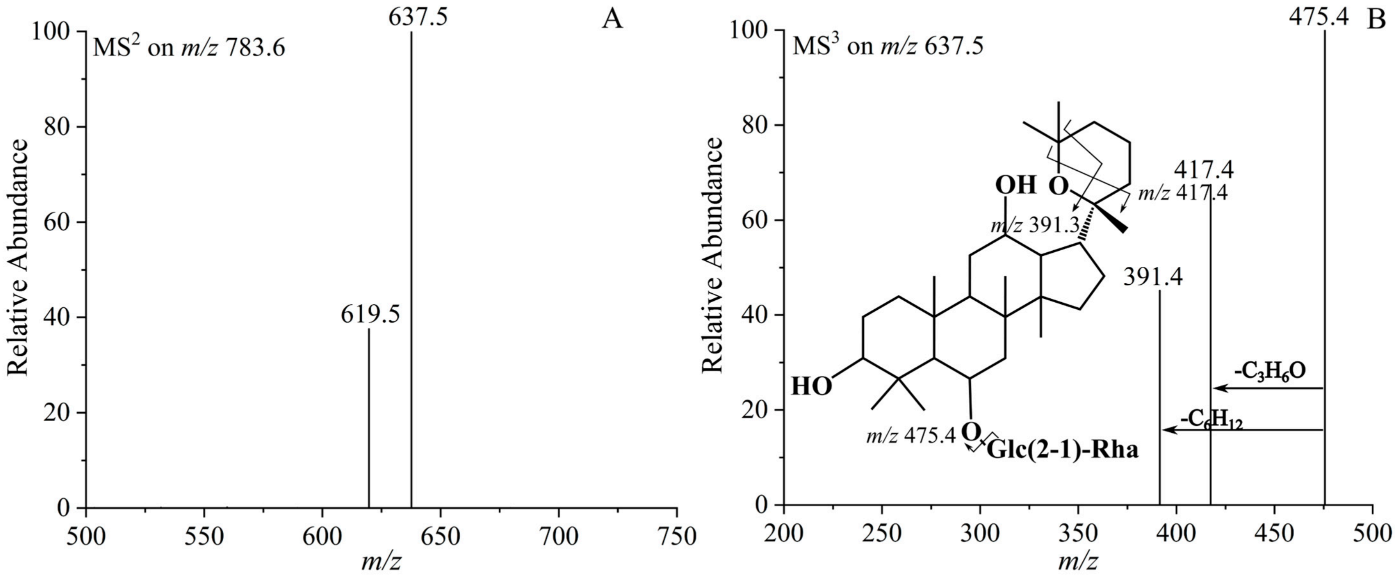


**Figure S12.** MS$^2$ spectrum of the $[M{-}H]^-$ ion at $m/z$ 783.6 (A), fragmentation pathways, and MS$^3$ spectrum of the product ion at $m/z$ 637.5 (B) from ginsenoside (20*R*,25)-epoxy-Rg2.

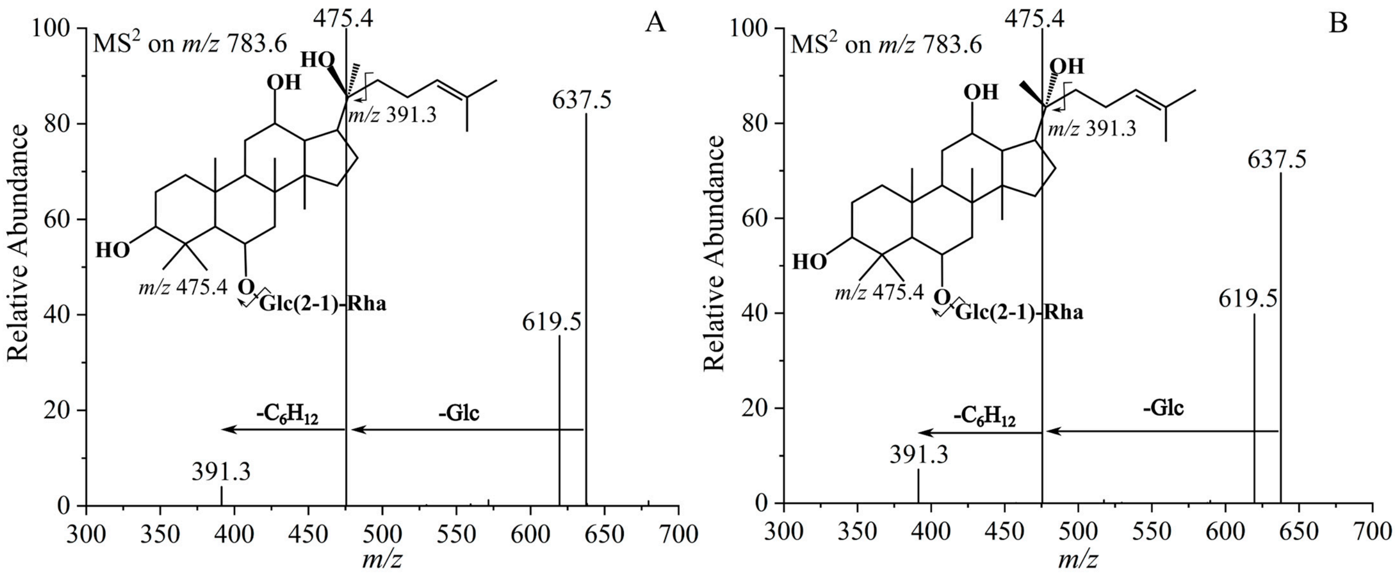


**Figure S13.** MS$^2$ spectra of the $[M-H]^-$ ion at *m/z* 783.6 from ginsenosides (20*S*)-Rg2 (A) and (20*R*)-Rg2 (B).

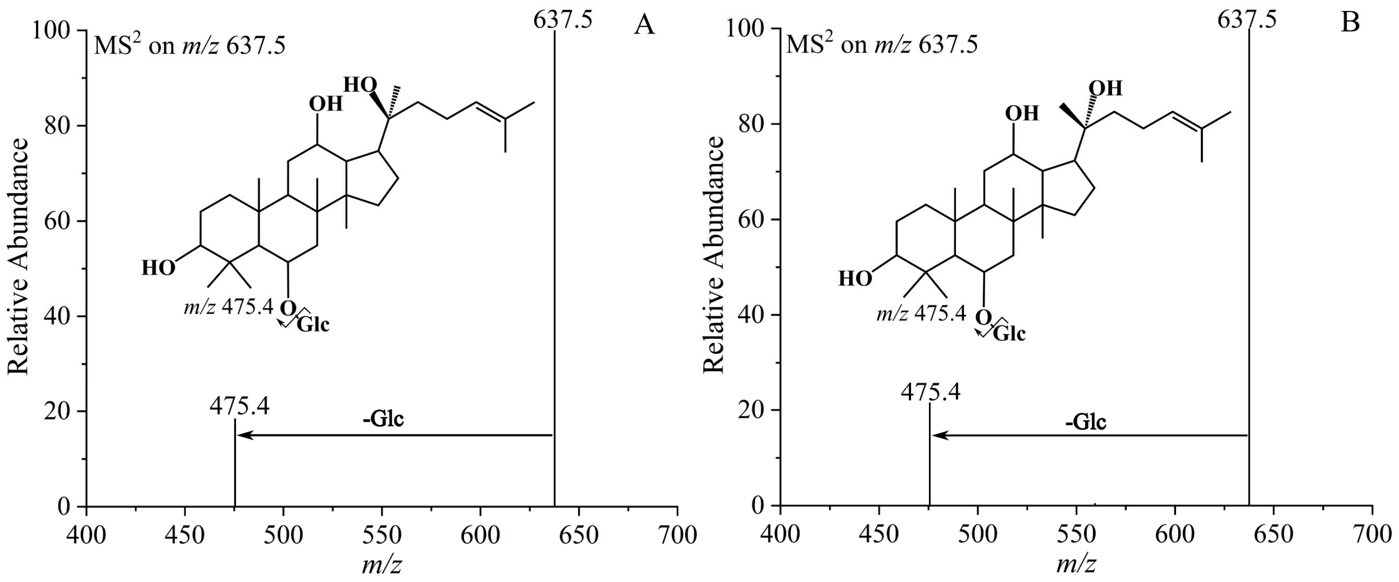


**Figure S14.** MS$^2$ spectra of the $[M-H]^-$ ion at *m/z* 637.5 from ginsenosides (20*S*)-Rh1 (A) and (20*R*)-Rh1 (B).

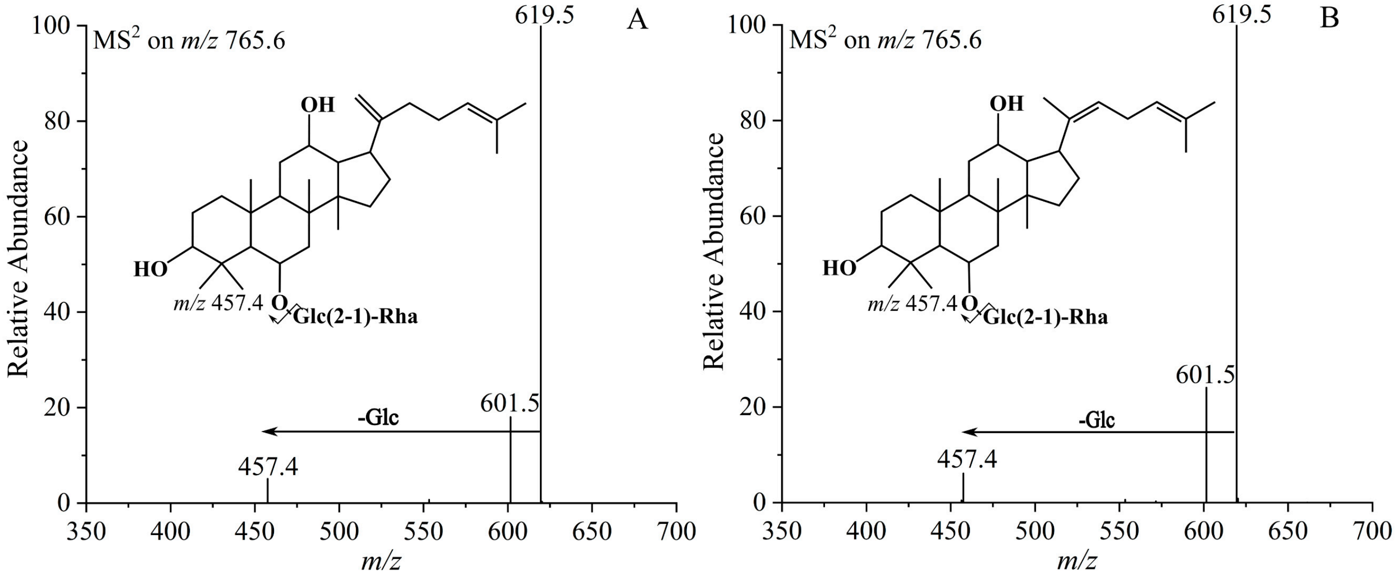


**Figure S15.** $MS^2$ spectra of the $[M{-}H]^-$ ion at *m/z* 765.6 from ginsenosides Rg6 (A) and F4 (B).

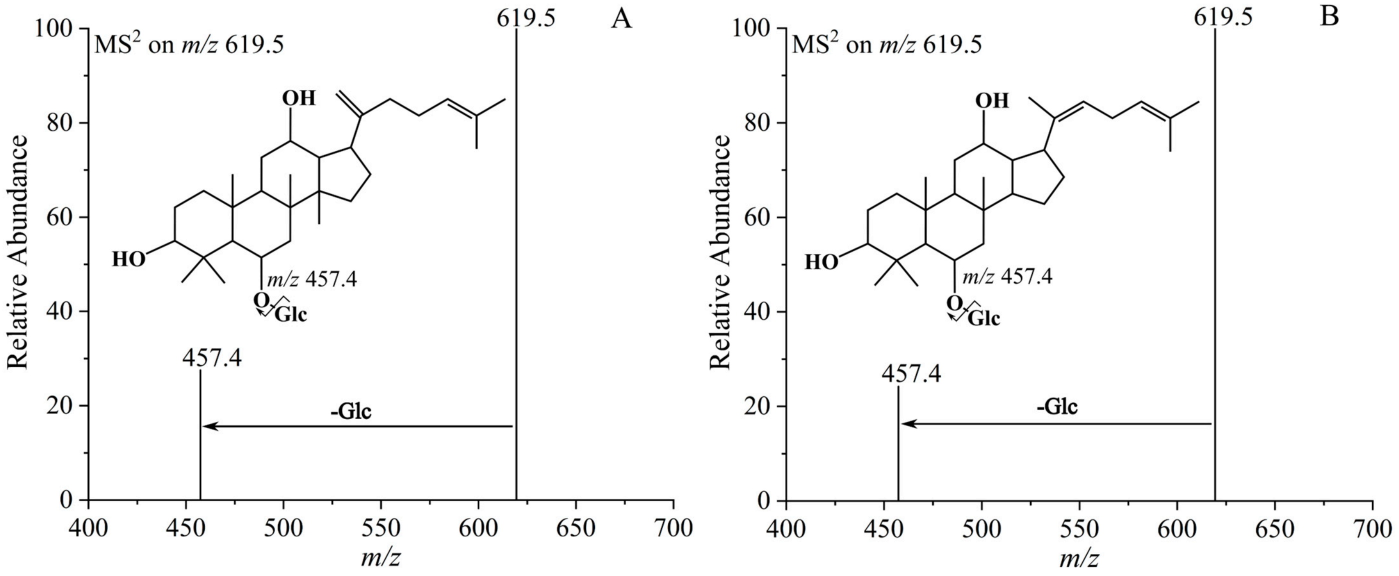


**Figure S16.** $MS^2$ spectra of the $[M{-}H]^-$ ion at *m/z* 619.5 from ginsenosides Rk3 (A) and Rh4 (B).
